# Propylene Metathesis over Molybdenum Silicate Microspheres with Dispersed Active Sites

**DOI:** 10.1021/acscatal.3c02045

**Published:** 2023-09-20

**Authors:** David Skoda, Ran Zhu, Barbora Hanulikova, Ales Styskalik, Vit Vykoukal, Petr Machac, Lucie Simonikova, Ivo Kuritka, Claude Poleunis, Damien P. Debecker, Yuriy Román-Leshkov

**Affiliations:** † Centre of Polymer Systems, Tomas Bata University in Zlin, tr. Tomase Bati 5678, Zlin CZ-76001, Czech Republic; ‡ Department of Chemical Engineering, Massachusetts Institute of Technology (MIT), 77 Massachusetts Avenue, Cambridge, Massachusetts 02139, United States; § Department of Chemistry, Faculty of Science, Masaryk University, Kotlarska 2, Brno CZ-61137, Czech Republic; ∥ Central European Institute of Technology, Masaryk University, Kamenice 5, Brno CZ 62500, Czech Republic; ⊥ Institute of Condensed Matter and Nanosciences (IMCN), Université catholique de Louvain (UCLouvain), Place Louis Pasteur 1, 1348 Louvain-la-Neuve, Belgium

**Keywords:** molybdenum silicate, olefin metathesis, nonaqueous, microspheres, catalyst, propylene

## Abstract

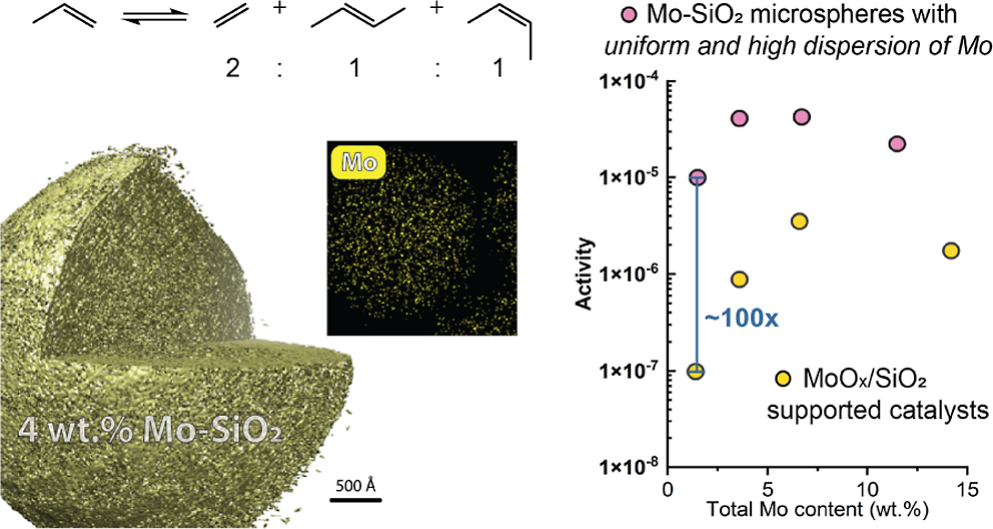

In this work, we demonstrate that amorphous and porous molybdenum silicate microspheres are highly active catalysts for heterogeneous propylene metathesis. Homogeneous molybdenum silicate microspheres and aluminum-doped molybdenum silicate microspheres were synthesized via a nonaqueous condensation of a hybrid molybdenum biphenyldicarboxylate-based precursor solution with (3-aminopropyl)triethoxysilane. The as-prepared hybrid metallosilicate products were calcined at 500 °C to obtain amorphous and porous molybdenum silicate and aluminum-doped molybdenum silicate microspheres with highly dispersed molybdate species inserted into the silicate matrix. These catalysts contain mainly highly dispersed MoO_*x*_ species, which possess high catalytic activity in heterogeneous propylene metathesis to ethylene and butene. Compared to conventional silica-supported MoO_*x*_ catalysts prepared via incipient wetness impregnation (MoIWI), the microspheres with low Mo content (1.5–3.6 wt %) exhibited nearly 2 orders of magnitude higher steady-state propylene metathesis rates at 200 °C, approaching site time yields of 0.11 s^–1^.

## 1 Introduction

Olefin metathesis is a versatile reaction for rearranging the C=C bonds of olefins, with a wide range of applications in the chemical, polymer, and pharmaceutical industries.^1^ In particular, the on-purpose production of propylene from the metathesis of ethene and 2-butene is an important technology to meet increasing propylene demand.^1^ Olefin metathesis catalysts, largely based on molecular transition metal (Mo, W, Re, and Ru) alkylidene complexes (M=CHR), have been developed for organic synthesis and polymer production.^1,2^ In parallel, heterogeneous catalysts used industrially for the metathesis of light olefins are typically based on supported W or Mo oxides.^3–6^ For example, a WO_*x*_/SiO_2_ catalyst is used for propylene production in the Phillips Triolefin Process;^7^ however, high reaction temperatures (>300 °C) are required for appreciable propylene yields (>40%).^5^ On the other hand, supported MoO_*x*_ catalysts are active for alkene metathesis under milder conditions (room temperature to 250 °C) but are less tolerant to process impurities.^8,9^

The olefin metathesis catalytic cycle over heterogeneous metal oxides is believed to occur via the widely accepted Chauvin mechanism involving metal alkylidene species.^2,10,11^ It is hypothesized that the active metal alkylidene sites are formed in situ when the alkene contacts the metal oxide precatalyst.^2,10,12^ Although the actual mechanism for this initiation process is not fully understood, it has been observed that the structure, oxidation state, and coordination of the metal oxide species have a marked impact on the catalyst’s performance. Based on experimental studies, several routes have been proposed to explain surface alkylidene formation. For instance, for silica-supported molybdenum oxide, Amakawa et al. postulated that Mo(VI) sites exclusively present in the freshly calcined catalyst are reduced by propylene into Mo(IV) species, and then Mo(VI)=CHR surface species are formed from the oxidative addition of another propylene molecule.^10,13^ Computational work by Handzlik et al. showed that surface silanol groups interact with the dioxo Mo(VI) or mono-oxo Mo(IV) species and that this interaction enables alkene protonation to form directly Mo(VI) alkoxy or Mo(VI) alkyl species, respectively.^14^ Further deprotonation of the alkoxy and alkyl ligands results in a Mo(IV) site and a Mo(VI) alkylidene species, respectively. Indeed, the geometry of the Mo sites, together with the local structure of the silica support, significantly influences the reactivity and the initiation mechanism. Research aimed at identifying the active sites of molybdenum-based silica catalysts and understanding their mode of activation has intensified in recent years.^8,9,15–19^ What unambiguously emerges from the scientific literature is that the catalyst preparation methods leading to molybdenum-silicate metathesis catalysts have a tremendous impact on the level of performance that can be attained. Classical preparation methods mostly revolve around the impregnation of a Mo precursor onto a preformed support, followed by thermal treatments.^9,20–23^ Several studies, however, have shown that alternative preparation methods (e.g., thermal spreading and flame spray pyrolysis) can lead to significantly more active catalysts.^3,12,24^ Of particular interest are nonhydrolytic sol–gel (NHSG) approaches, which are known to give access to well-defined, porous, heterogeneous catalysts with homogeneously dispersed active sites for various catalytic reactions.^25,26^

In NHSG, a nonaqueous medium is used to drive the sol–gel process exclusively via nonhydrolytic polycondensation reactions (i.e., there is no hydrolysis step). This leads to a higher degree of condensation, deeper cross-linking, and thus better homogeneity with a high content of M–O–Si linkages (M = metal). Moreover, phase separation of oxides, usually caused by the rapid hydrolysis of metal precursors (i.e., M–OH species formation) followed by M–OH group condensation to M–O–M bridges, is prevented.^26,27^ Hence, a more homogeneous dispersion of active sites compared to conventional catalysts prepared by an aqueous sol–gel approach is achieved. The formation of isolated species that are tightly incorporated into a porous silicate matrix is of great importance when trying to prepare highly active species for many catalytic reactions. The high degree of condensation in the gel and the adoption of low surface tension solvents also facilitate the drying and the obtaining of mixed oxides with an advantageous texture, similar to that of aerogels. Knowing this, it is unsurprising that NHSG has already proven very effective in preparing highly active olefin metathesis catalysts.^27–31^

We have recently introduced a facile method for preparing homogeneous molybdenum silicate microspheres and shown their application as heterogeneous catalysts for epoxidation reactions.^32^ The synthesis based on the NHSG condensation of a hybrid molybdenum biphenyldicarboxylate-based precursor solution (labeled as Mo-Bpdc) with (3-aminopropyl)triethoxysilane allows for a homogeneous distribution of highly dispersed molybdenum species within a silica matrix after calcination. These findings prompted us to investigate the potential of such Mo-based catalysts in the olefin metathesis reaction. Herein, we report the high activity of molybdenum-silicate-based spherical catalysts with different molybdenum loadings for the propylene self-metathesis reaction. The improved performance in metathesis rates of the microsphere catalysts compared with the catalysts prepared via incipient wetness impregnation (IWI) is associated with the high proportion of isolated MoO_*x*_ active sites, as demonstrated through a thorough characterization survey.

## 2 Experimental Section

### 2.1 Chemicals

Bis(acetylacetonato)dioxomolybdenum(VI) (MoO_2_(acac)_2_, 99%), biphenyl-4,4′-dicarboxylic acid (H_2_Bpdc, 97%), (3-aminopropyl)triethoxysilane (H_2_N(CH_2_)_3_Si(OC_2_H_5_)_3_, APTES, 98%), aluminum(III) acetylacetonate (99%), and the silica support (Davisil grade 646, pore size 150 Å, 300 m^2^ g^–1^) were supplied from Sigma-Aldrich. *N*,*N*′-dimethylformamide (DMF, anhydrous) was purchased from VWR (Avantor). Ammonium molybdate tetrahydrate (≥99%) was purchased from Fluka. Silicon carbide (46 grit) was supplied by Alfa Aesar. All gases used for the synthesis of silica-supported molybdenum oxide catalysts, in situ spectroscopy, and reactivity experiments were purified to reduce the concentration of impurities. Specifically, helium (UHP 5.0 grade) and propylene (>99.95%, electronic grade) were purchased from Airgas and purified by passing through individual traps containing 3 Å molecular sieves (4–8 mesh) from Sigma-Aldrich and preactivated 5 × 3 mm R3-11G tablets from Research Catalysts. Air supply was house air sequentially purified with a Wilkerson modular compressed air filter from McMaster-Carr, an FID tower (NM Plus 1350 FID Tower) from VICI DBS, and an indicating moisture trap from Restek.

### 2.2 Catalyst Synthesis

#### 2.2.1 Synthesis of Mo-Bpdc-Si Solid Precursors

Bis(acetylacetonato)dioxomolybdenum(VI) (MoO_2_(acac)_2_) (desired mass for each Mo loading, Table S1) was dissolved in 50 mL of DMF in a Teflon container. After the dissolution of the molybdenum precursor, biphenyl-4,4′-dicarboxylic acid (H_2_Bpdc) (0.24 g, 1.00 mmol) was added, and the container was closed tightly and placed in a microwave reactor (ERTEC Magnum II; 600 W; 2.45 GHz). Microwave power was set to 50% (300 W), and the reaction mixture was heated to 160 °C under microwave irradiation for 30 min. The temperature of 160 °C was reached in ca. 8 min. The hybrid intermediate product of the microwave-assisted reaction was labeled Mo-Bpdc. Once the reaction mixture was cooled down to room temperature, the yellow transparent solution of Mo-Bpdc precursor was slowly dropped into a stirred solution of (3-aminopropyl)triethoxysilane (APTES) (1.12 g, 5.05 mmol) in 50 mL DMF. The mixture became cloudy during the mixing, and a white precipitate was formed. As confirmed by gas chromatography–mass spectrometry (GC–MS), the condensation reaction between Mo-Bpdc hybrid intermediate and APTES proceeds with ethanol elimination (Figure S1). Once the whole volume of the Mo-Bpdc precursor was added to the APTES solution, the mixture was continuously stirred at room temperature for 4 h. Then, the precipitate formed within the condensation of Mo-Bpdc precursor and APTES (labeled as Mo-Bpdc-Si) was separated by centrifugation (6000 rpm, 5 min), washed with DMF, and dried in an oven at 80 °C overnight. The amount of each separated hybrid Mo-Bpdc-Si solid precursor was about 0.7 g.

#### 2.2.2 Synthesis of Al-Mo-Bpdc-Si Solid Precursors

Aluminum(III) acetylacetonate (0.324 g, 1.002 mmol) was added to a stirred solution of MoO_2_(acac)_2_ (0.164 g, 0.502 mmol) in 60 mL of DMF. After the dissolution of both molybdenum and aluminum precursors, the following steps were followed in the same manner as the Mo-Bpdc-Si solid precursors described above.

#### 2.2.3 Thermogravimetric Analysis and Calcination Process

Thermogravimetric analyses of the solid precursors (Mo-Bpdc-Si and Al–Mo-Bpdc-Si) were performed on a Setaram LabSys Evo instrument with a TG/DSC sensor in an air atmosphere (heating ramp 5 °C min^–1^, up to 1000 °C, air flow 60 mL min^–1^). The decomposition and release of the Bpdc linker and other organic moieties (e.g., aminopropyl group) were recorded on thermogravimetric curves (Figure S2). Based on the results from the thermogravimetric analyses, all solid precursors were calcined in a Nabertherm LE 4/11/R6 tubular furnace at 500 °C (heating ramp rate of 5 °C min^–1^) for 3 h under flowing air to generate inorganic Mo–SiO_2_-based samples, which were utilized as catalysts for propylene metathesis. Calcined samples were labeled as 11Mo–SiO_2_, 7Mo–SiO_2_, 4Mo–SiO_2_, 2Mo–SiO_2_, and Al11Mo–SiO_2_ to reflect the wt % of Mo in the samples.

#### 2.2.4 Synthesis of Silica-Supported Molybdenum Oxide Catalysts (MoIWI)

Silica-supported molybdenum oxide catalysts were prepared as benchmarks according to published procedures.^22,23^ Prior to the synthesis, the silica gel support was heated at 500 °C (3 °C min^–1^) for 3 h under an air flow (60 mL min^–1^) and then sieved to select particle sizes in the 40–60 mesh range. Depending on the desired Mo loading, different amounts of ammonium molybdate tetrahydrate were dissolved in Milli-Q water (4 mL). The solution was added dropwise to 2.0 g of SiO_2_ (calcined, 40–60 mesh) support. The slurry was stirred until no obvious color gradient was observed and then dried at room temperature for 6 h. Next, the slurry was dried at 90 °C (1 °C min^–1^) for 3 h and calcined at 400 °C (1 °C min^–1^) under an air flow (60 mL min^–1^) for 3 h. After calcination, the catalyst was sieved again to collect the particles in the 40–60 mesh size range.

### 2.3 Characterization Techniques

#### 2.3.1 Chemical and Elemental Analyses

The GC–MS analysis of reaction byproducts was performed on an ISQ Series single-quadrupole mass spectrometer coupled to a Trace 1300 gas chromatograph. The gas chromatograph was equipped with a Rxi-5 ms column (30 m, 0.25 mm, film thickness 0.25 μm), and the temperature program was set as follows: 80 to 240 °C with the ramp at 10 °C min^–1^, held at 240 °C for 1 min. Split injection mode (60 mL min^–1^, split ratio 60) was set with the inlet and transfer line temperatures of 220 °C and the EI source at 220 °C (ionization energy: 70 eV, emission current: 30 μA). The content of molybdenum, aluminum, and silicon elements in the Mo–SiO_2_ samples and MoIWI catalysts was determined by ICP–OES spectroscopy performed on an iCAP 6500 spectrometer (molybdenum spectral line λ = 202.030 nm, aluminum spectral line λ = 394.401 nm, and silicon spectral line λ = 250.690 nm). All instruments are part of the Thermo Scientific portfolio.

#### 2.3.2 XRD Diffraction Analysis

The powder XRD patterns were recorded on a Rigaku MiniFlex 600 diffractometer equipped with a Co Kα (λ = 1.7903 Å) X-ray tube (40 kV, 15 mA). Data processing was performed with Rigaku PDXL2 software.

#### 2.3.3 Infrared and Raman Spectroscopy

The FTIR spectra of prepared Mo-Bpdc-Si-based solid precursors and Mo–SiO_2_-based microspheres were recorded on a Thermo Nicolet 6700 spectrometer using an ATR technique with the diamond crystal (resolution 2 cm^–1^, 4000–400 cm^–1^) (Figures S3 and S4). Raman spectroscopy was performed on a Nicolet DXR Thermo Raman microscope equipped with a He–Ne laser with an excitation wavelength of 780 nm. The spectra were recorded from 2000 to 50 cm^–1^ under standard ambient conditions.

The in situ transmissive FTIR spectra were collected with a Bruker Vertex 70 spectrometer with a liquid N_2_-cooled Hg–Cd–Te (MCT) detector. The high-temperature transmission IR cell (HTC-3-XXX) from Harrick Scientific was sealed with two KBr windows (32 × 3 mm). The inlet of the IR cell was connected to a Schlenk line setup described elsewhere.^33^ Each spectrum accumulated 128 scans at 4 cm^–1^ resolution and an aperture setting of 4 mm between 4000 and 400 cm^–1^. Approximately 7 mg of sample was pressed into 7 mm diameter pellets, secured in a stainless-steel pellet holder (038-141-2) from Harrick Scientific, and loaded into the IR cell. Prior to each experiment, the sample pellet was heated to 400 °C (3 °C min^–1^) for 3 h and cooled to 150 °C with flowing air (60 mL min^–1^). Spectra for samples after heat treatment were collected relative to a baseline recorded with an empty cell at 150 °C under flowing air (60 mL min^–1^). Then, the IR cell was evacuated until pressure was steady at <10^–5^ Torr. Pyridine (anhydrous, >99.8 wt %, further purified via freeze–pump–thaw cycles) from MilliporeSigma was sequentially introduced to the IR cell until chemisorbed pyridine IR features reached a steady state. After each dose (0.1–1.0 μmol per dose), a spectrum was recorded relative to a baseline obtained with an empty cell at 150 °C under a high vacuum (<10^–5^ Torr). The spectra shown in this study represent the catalyst surface saturated with chemisorbed pyridine.

For propylene adsorption, the sample pellet was heated under flowing air (60 mL min^–1^) at 400 °C (3 °C min^–1^) for 3 h and purged with helium (100 mL min^–1^) at 300 °C for 1 h. The sample pellet was then exposed to 50 mL min^–1^ 20% propylene balanced with helium at 200 °C until no obvious changes were observed in the last two FTIR spectra. The sample pellet was further purged with 100 mL of min^–1^ helium to remove excess propylene in the analysis chamber. The in situ FTIR spectra shown here represent samples at steady-state post propylene adsorption.

The Raman spectra of MoIWI were acquired by using a Renishaw Invia Reflex Micro Raman equipped with a 532 nm laser. Prior to the spectral acquisition, samples (50–60 mg) were dehydrated ex situ in a Raman High Temperature Reaction chamber (Harrick Scientific, HVC-MRA-5). Samples were calcined at 400 °C (3 °C min^–1^, hold for 3 h) and cooled to room temperature under flowing purified house air (50 mL min^–1^). Then, the cell was sealed using 2-way bellows sealed valves (Swagelok, SS-4H) and transferred into the Raman sample chamber. Spectra were obtained at a resolution of 2 cm^–1^ at room temperature using a laser power of 25 mW with an accumulation of 32 scans.

#### 2.3.4 In Situ UV–Vis Spectroscopy

In situ UV–vis spectra were acquired using a Cary 5000 UV–vis–NIR spectrophotometer using a DiffusIR environmental chamber (162-4200) from PIKE Technologies sealed with a SiO_2_ disk (32 × 3 mm). Each spectrum was acquired at a 600.0 nm min^–1^ scan rate and a data interval of 1.0 nm between 800 and 200 nm. All UV–vis spectra were collected relative to a baseline of BaSO_4_ (99 wt %) from MilliporeSigma under standard ambient conditions. The samples were loaded into the ceramic cup (162-4251) from PIKE Technologies and placed into the chamber. Before collecting the UV–vis spectra, samples were heated to 400 °C (3 °C min^–1^) for 3 h and cooled to 50 °C under flowing air (60 mL min^–1^). Diffuse reflectance measurements were converted to absorbance using the Kubelka–Munk function.^34^ The edge energy for direct allowed transitions was estimated by the intercept of a straight line fitted at the low-energy rise of a plot of [FR(∞)*h*υ]^2^ as a function of *h*υ (incident photon energy).^34,35^

#### 2.3.5 Temperature-Programmed Reduction with Hydrogen (H_2_-TPR)

H_2_-TPR was performed with a Micromeritics Autochem II 2920 unit equipped with a thermal conductivity detector. The microspheres (70–80 mg) were loaded in a U-shaped quartz reactor between two layers of quartz wool. The samples were pretreated under flowing 50 mL min^–1^ 5% O_2_ balanced with He at 400 °C (3 °C min^–1^) for 3 h and purged under flowing 50 mL min^–1^ He at 150 °C for 1 h. The analysis for the H_2_-TPR experiments was conducted in 10% H_2_ balanced with Ar (50 mL min^–1^) by heating up the microspheres in the range from 100 to 950 °C at 10 °C min^–1^. The measured H_2_ consumption was normalized to the mass of the sample.

#### 2.3.6 X-ray Photoelectron Spectroscopy

X-ray photoelectron spectroscopy (XPS) measurements were carried out on a Kratos Analytical Axis Supra spectrometer equipped with monochromated Al Kα radiation (1486 eV). The sample powders were deposited on double-sided copper tape attached to the sample holder. The pressure in the analysis chamber was ca. 10^–6^ Pa. The analyzed area was approximately 1.4 mm^2^, and the pass energy was set at 150 eV. The C 1s peak of carbon was fixed at 284.8 eV to set the binding energy scale.^36^ Data treatment was performed with the CasaXPS program (Casa Software Ltd., UK), and spectra were deconvoluted with the least-squares fitting routine provided by the software with a Gaussian/Lorentzian (85/15) product function after subtraction of a nonlinear baseline.^37^

#### 2.3.7 Microscopy

The scanning electron microscopy (SEM) was performed on a Nova NanoSEM (FEI) instrument operated at 5 kV and equipped with a Schottky field emission gun (FEG) electron source and TLD secondary-electron detector. The SEM energy dispersive X-ray (EDX) analyses were performed on an EDX spectrometer octane Plus (EDAX, AMETEK, Inc.) with an SDD detector. The transmission electron microscopy was performed with a high-resolution TEM Titan Themis 60–300 High Base (Thermo Fisher Scientific) operated at 300 kV and equipped with a high-brightness X-FEG electron source and a spherical aberration image (Cs)-corrector. The scanning TEM EDX (STEM–EDX) analyses were performed with a Super-X spectrometer (Thermo Fisher Scientific) equipped with four 30 mm^2^ windowless detectors. The STEM imaging was performed using a high-angular annular dark-field detector (Fishione), which provided an atomic Z-contrast. The sample for TEM was dispersed in methanol, and 4 μL of this suspension was dropped onto a QuantiFoil R 2/1300 mesh copper grid and dried by evaporation at standard ambient conditions. TEM tomography was performed on a Titan Krios transmission electron microscope equipped with a Volta phase plate, an energy filter, and a Gatan K2 direct electron camera. This microscope was operated at 300 kV and aligned for fringe-free imaging.

#### 2.3.8 Nitrogen Adsorption/Desorption

Nitrogen adsorption/desorption isotherms were collected at 77 K on a BELsorp Mini II (Japan). Before measurement, the samples were degassed in the BELsorp preparation unit at 100 °C for 19 h. The surface area was calculated using the multipoint Brunauer–Emmet–Teller (BET) method using at least five data points with relative pressures between 0.05 and 0.30.^38^ The total pore volume was measured at a *p*/*p*_0_ = 0.99.

#### 2.3.9 Solid State NMR Spectroscopy

^13^C CP TOSS, ^29^Si (both CP and direct excitation), and ^27^Al solid-state NMR spectra were measured on a Bruker Avance III HD 700 MHz spectrometer with a MAS DVT 700S4 BL4 N–P/H probe. Chemical shifts were referenced externally to ^29^Si δ [(Me_3_SiO)_8_Si_8_O_20_]: 11.72 ppm; ^13^C δ [adamantane]: 38.68 ppm; ^27^Al δ [[Al(H_2_O)_6_]^3+^ (aq solution)]: 0.0 ppm.

#### 2.3.10 Time of Flight Secondary Ion Mass Spectrometry

Chemical characterization of samples was carried out using a TOF-SIMS^5^ instrument (IONTOF GmbH, Münster, Germany). A pulsed Bi_5_^+^ metal ion source was used to produce a primary beam using an acceleration voltage of 30 kV. An AC target current of 0.08 pA with a bunched pulse width of less than 1 ns was used. Both positive and negative secondary ion species were analyzed. A raster of 128 × 128 data points over an area of 300 × 300 μm^2^ was used for spectra. The total primary ion beam dose for each analyzed area was kept below 3 × 10^10^ ions cm^–2^, ensuring static conditions. The lateral resolution of ∼3 μm and mass resolution *m*/Δ*m* > 4000 at 29 *m*/*z* were maintained for positive and negative spectra acquisition. Charge compensation was performed by an interlaced electron flood gun (*E*_k_ = 20 eV). All data analyses were carried out using the software supplied by the instrument manufacturer, SurfaceLab (version 6.8). Sample powders were pressed onto the adhesive part of the Postit papers.

### 2.4 Reactivity Measurements

The reactivity of catalysts for propylene metathesis was measured in a U-shape tubular pack bed reactor (SS304, 1/4 in. OD, 0.18 in. ID). Silicon carbide (SiC) was used as a diluent for the bed. Approximately 20 mg of sample was mixed with 100 mg of inert diluent SiC and packed between two layers of 150 mg of inert SiC and two quartz wool plugs (4–6 μm) from Technical Glass Products. A type-K thermocouple from Omega Engineering was placed on top of the catalyst bed, touching the upper quartz wool layer.

The reactor setup was leak-checked before each experiment. In a typical pretreatment procedure, the sample was treated at 400 °C (3 °C min^–1^) for 3 h and cooled to 270 °C under flowing air (50 mL min^–1^). Then, the sample was purged at 270 °C for 30 min and activated at 500 °C (2 °C min^–1^) for 3 h under flowing helium (100 mL min^–1^). Finally, the sample was cooled to the reaction temperature under flowing helium (50 mL min^–1^). Heating was provided by a home-built reaction furnace, which consists of high-temperature, dual-element heating tape (DHT051060LD) from Omega Engineering wrapped around a tube (SS304, 2-1/2 in. OD, 2 in. ID, 6 in. long). The furnace was wrapped with a 1-1/2 in.-thick ceramic fiber insulation sheet from McMaster-Carr and an aluminum sheet (0.016 in. thick) to maintain the temperature inside the furnace. After loading the reactor into the furnace, the bottom and top of the furnace were sealed with 1/2-in.-thick ceramic fiber insulation sheets. The furnace was controlled by a Cole-Parmer temperature controller (Digi-Sense 89000). Transfer lines from the outlet of the individual traps to the outlet of the reactor were heat traced to >90 °C to remove olefin residuals inside the reactor system. The background reactivity of the reactor system was measured with an empty reactor solely consisting of inert SiC and SiO_2_. Traceable C6 olefins (mainly methyl-pentene isomers) from the traps were observed with no ethylene, *trans*-2-butene, or *cis*-2-butene. The amount of C6 olefins from the traps was independent of reactor temperature and was subtracted when estimating the rate of propylene dimerization.

The reactor effluent was analyzed by online gas chromatography (GC; Shimadzu GC-2014) equipped with an Agilent HP-PLOT Al_2_O_3_–S (30 m × 0.25 mm) column and a flame ionization detector. Reaction products were periodically collected and analyzed offline using GC–MS (Agilent 7820A and 5977B MSD). Products were identified by matching GC retention times with known standards and comparing MS fragmentation patterns to those available in NIST libraries. All products were quantified by means of a flame ionization detector calibrated against known standards.

In this study, all reported rates were steady-state rates. We defined the rate of propylene metathesis as the sum of the production rates of ethylene, *trans*-2-butene, and *cis*-2-butene. The rate of propylene dimerization was defined as the sum of the production rates of all C6 olefins. The catalyst mass normalized rate of reaction (*r*), molybdenum content normalized site time yield (STY), and propylene conversion were calculated according to eqs 1–3, respectively. The mass balance of all data points is close to the expected uncertainties of the flow rates.
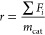
1
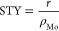
2
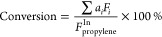
3where *F*_*i*_ is the molar flow rate of species *i*; *i* stands for each product from the corresponding reaction; *m*_cat_ is the mass of the catalyst; ρ_Mo_ (mole of Mo per gram of catalyst) is the total Mo molar density of the sample; *a*_*i*_ is the stoichiometric number of propylene for product *i*, e.g., 1 for ethylene and *cis*-/*trans*-2-butene, and 2 for C6 olefins; and *F*^In^_propylene_ is the flow rate of propylene into the reactor.

## 3 Results and Discussion

### 3.1 Characterization of the Catalysts

In this work, we implemented a NHSG approach to prepare porous Mo–SiO_2_ microspheres with different molybdenum contents and, in some cases, with an aluminum dopant. The aluminum doping was investigated to modify the surface acidity and introduce a higher number of Brønsted acid sites. Such modification was intended to possibly improve the catalytic properties of aluminum–molybdenum silicate catalysts for the olefin metathesis reaction.^6,39^ All Mo-Bpdc-Si-based solid precursors and Mo–SiO_2_-based microspheres exhibited no diffraction maxima in their powder X-ray diffraction patterns (Figures S5 and S6), confirming their amorphous character.

**Figure 1:**
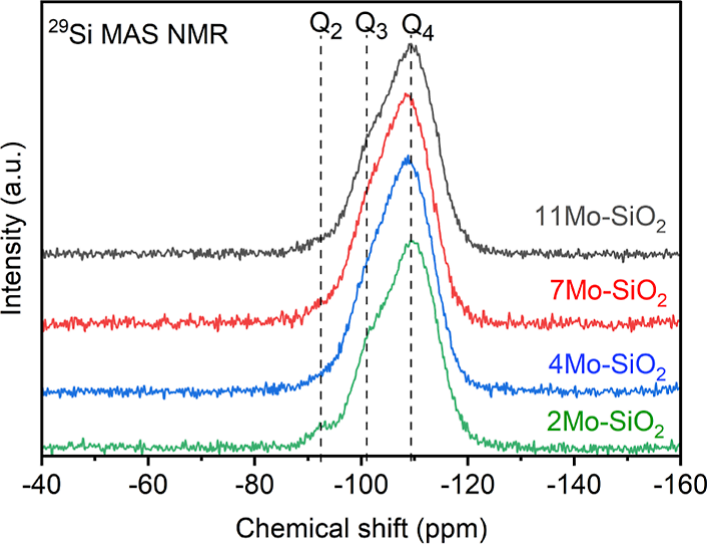
^29^Si MAS NMR spectra of molybdenum-silicate catalysts.

The ^29^Si MAS NMR spectra of Mo–SiO_2_ microspheres exhibited signals corresponding to Q_*n*_ sites in silica-based materials (Figure 1). This observation, together with the lack of signals associated with T_2_ and T_3_ sites^40^ in the ^29^Si MAS NMR spectra (Figure S7), implies a successful transformation of the organosiloxanes in Mo-Bdpc-Si solid precursors to fully inorganic silicates upon calcination. Features denoted as Q_4_, Q_3_, and Q_2_, which show the signals of Si(OSi)_4_, (MoO)Si(OSi)_3_/HO–Si(OSi)_3_, and (MoO)_2_Si(OSi)_2_/(HO)_2_Si(OSi)_2_ species,^40–42^ respectively, were similar to each other for all Mo–SiO_2_ microspheres. As expected, since the MAS NMR spectra were measured without cross-polarization, the highest intensity was obtained for Q_4_ sites (−110 ppm).^42,43^ The signal associated with the Q_3_ (MoO)Si(OSi)_3_/HO–Si(OSi)_3_ site was found at −101 ppm. The ^29^Si CPMAS NMR spectra of prepared Mo–SiO_2_ and Al11Mo–SiO_2_ microspheres (Figures S8 and S9) exhibited identical characters, with the most intense signal of Q_3_ sites at −101 ppm due to the coupling with the hydroxyl protons of silanol groups.^44^ The ^27^Al MAS NMR spectrum of Al11Mo–SiO_2_ microspheres (Figure S9) showed a sharp signal at 0 ppm associated with the presence of six-coordinated aluminum species.^45^

**Figure 2:**
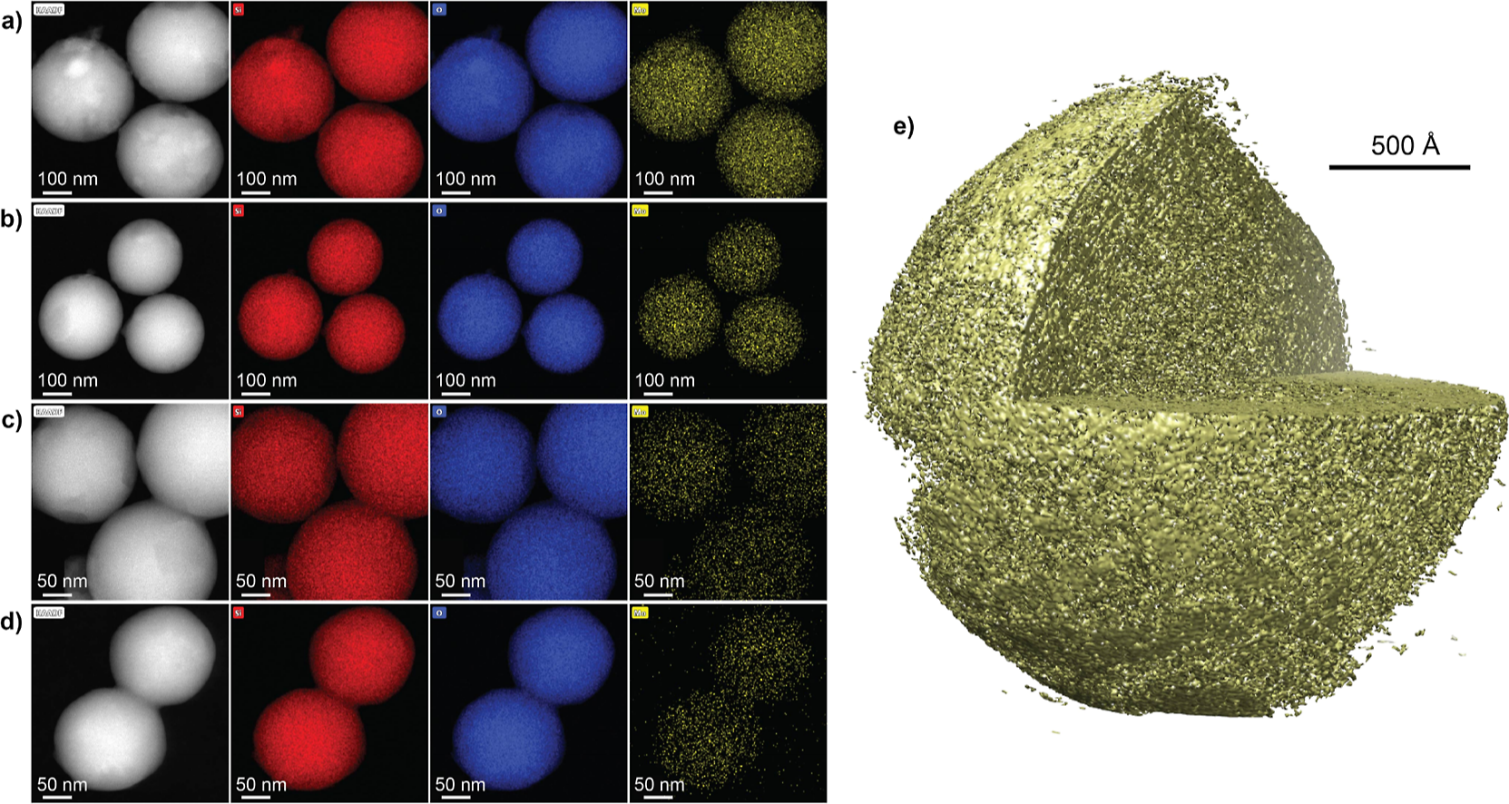
STEM-EDS elemental maps of (a) 11Mo–SiO_2_, (b) 7Mo–SiO_2_, (c) 4Mo–SiO_2_, and (d) 2Mo–SiO_2_. Color codes: red—Si, blue—O, and yellow—Mo. (e) 3D TEM tomography visualization of the 4Mo–SiO_2_ microsphere.

SEM and TEM imaging (Figures S10–S12) revealed that the Mo–SiO_2_ materials have a spherical morphology. The size of the microspheres decreased with the Mo content. The average sizes (Figure S13) of 11Mo–SiO_2_, 7Mo–SiO_2_, 4Mo–SiO_2_, and 2Mo–SiO_2_ were 313 ± 21, 298 ± 25, 225 ± 26, and 175 ± 22 nm, respectively. The STEM-EDS (Figure 2a–d) elemental maps showed that the distributions of silicon, oxygen, and molybdenum elements in the microspheres were homogeneous. This was also observed for Al in the Al11Mo–SiO_2_ sample (Figure S14). Detailed 3D TEM tomography of 4Mo–SiO_2_ indicates a microsphere with a homogeneous porous character within its entire volume (Figure 2e).

The elemental compositions of the microspheres were studied by ICP–OES and EDX methods (Table 1 and Tables S2 and S3). The results from the ICP–OES are in good agreement with the nominal compositions of the precursor gels (Table 1) and with the values of Mo and Si contents found by SEM–EDX mapping (Figures S15–S19). The Si/Mo weight ratios measured experimentally closely match the Si/Mo weight ratios calculated from the amounts of reaction precursors, highlighting excellent control over catalyst composition and quantitative condensation between molybdenum and silicon precursors.

**Table 1:** Molybdenum and Silicon Content Values by Different Methods and Si/Mo Weight Ratios

sample	Nominal Si/Mo weight ratio^a^	ICP–OES Si/Mo wt % ratio	ICP–OES [wt %]		EDX^b^ [wt %]		XPS [wt %]	
			Si	Mo	Si	Mo	Si	Mo
11Mo–SiO_2_	3.0	3.0	34.4	11.5	37.7	10.5	42.7	6.26
7Mo–SiO_2_	6.1	5.7	37.9	6.65	42.2	6.72	48.1	3.50
4Mo–SiO_2_	11.9	10.9	39.8	3.62	43.3	3.65	50.1	2.29
2Mo–SiO_2_	23.0	23.0	42.5	1.70	36.7	1.59	49.7	0.79

a Based on the initial weight of molybdenum and silicon precursors.

b The EDX spectra and elemental contents are given in the Supporting Information (Figures S15–S19, Table S2).

The surface composition was studied by XPS (Table 1, Table S2). In comparison to the bulk composition measured by ICP–OES (Tables 1 and S3), the values of the Mo concentration on the surface of the microspheres are lower. This could be due to the faster inorganic polycondensation reactions around Mo centers during synthesis, resulting in a surface that is relatively poorer in Mo than the interior of the walls. In the case of Al11Mo–SiO_2_ microspheres, this effect appears to be less pronounced, and both surface molybdenum and aluminum content more closely match that of the bulk (Figure S19, Tables S2 and S3). XPS (Figures S20–S29) was also employed to characterize the oxidation states of Mo on the surface of the microspheres. The results of XPS (Figures S30 and S31) showed the presence of Mo (VI) and Mo (V) in all Mo–SiO_2_ microspheres, which are in good agreement with the work by Lin et al. describing molybdenum incorporated in SBA-based silica.^46^

Nitrogen adsorption/desorption resulted in characteristic isotherms for slit-shaped pores (Figure S32).^47^ The surface area values determined by the BET method as well as total pore volume and micropore volume numbers are listed in Tables 2 and S4. All samples exhibited a high surface area above 300 m^2^ g^–1^, which increased with the decreasing content of Mo. The pore size distribution determined by the NLDFT method showed a mostly microporous character with a pore diameter of 1.2 nm. The hysteresis loops observed on N_2_ adsorption/desorption isotherms can be most likely assigned to the tensile strength effect^48^ (Figure S32).

**Table 2:** Surface Area and Total Pore Volume Values

samples	SA_BET_ [m^2^ g^–^^1^]	*V*_total_ [cm^3^ g^–^^1^]	band edge energy [eV]
11Mo–SiO_2_	322	0.24	4.07
7Mo–SiO_2_	367	0.25	4.08
4Mo–SiO_2_	376	0.30	4.09
2Mo–SiO_2_	400	0.34	4.03

Diffuse reflectance UV–vis spectroscopy performed after dehydration indicates that the *E*_g_ values ranged from 4.03 to 4.09 eV (Table 2, Figure S33). These values indicate a high proportion of isolated MoO_4_ units present in the catalysts.^35,49^ The Raman spectra (Figure S34) of all catalysts recorded under ambient conditions showed no peaks at 665 or 815 cm^–1^, which are typically assigned to MoO_3_ crystalline structures.^35^ In the case of MoO_*x*_/SiO_2_ catalysts prepared via the IWI method (MoIWI) (Table S5 and Figure S35), Raman spectra and edge energy of dehydrated samples exhibited oligomerized MoO_*x*_ species above Mo loadings of 3.6 wt % and crystalline MoO_3_ species above Mo loadings of 6 wt %.

Time-of-flight secondary ion mass spectrometry (ToF-SIMS) provided a further description of the molybdenum species present at the outermost surface of the catalysts.^12,50,51^ All relevant Mo-containing anions that have been detected and quantified are listed in Table S6. The total proportion of Mo-containing clusters from Mo–SiO_2_-based microspheres (i.e., the sum of the intensity for each Mo-containing anion normalized by the total count) expectedly increased with Mo loading (Figure S36). In the case of MoO_*x*_/SiO_2_ prepared via the IWI method (MoIWI), the total proportion of Mo-containing clusters was significantly higher (Figures S36 and S37). Interestingly, ToF-SIMS allows detecting Mo-based clusters with different Mo nuclearities. Detected ions could be pooled according to the nuclearity of Mo as follows: (1) ions with one Mo atom (MoO_3_^–^, MoO_4_^–^, and MoO_5_Si^–^), and (2) ions with multiple Mo atoms (Mo_2_O_6_^–^, Mo_2_O_7_^–^, Mo_2_O_8_Si^–^, and Mo_3_O_9_^–^). Here, the relative intensities of clusters with one Mo atom are useful proxies of the MoO_*x*_ dispersion. On the contrary, a high proportion of multiple Mo atom clusters indicates the presence of more condensed species such as poly molybdates or crystalline MoO_3_.^12,31^

**Figure 3:**
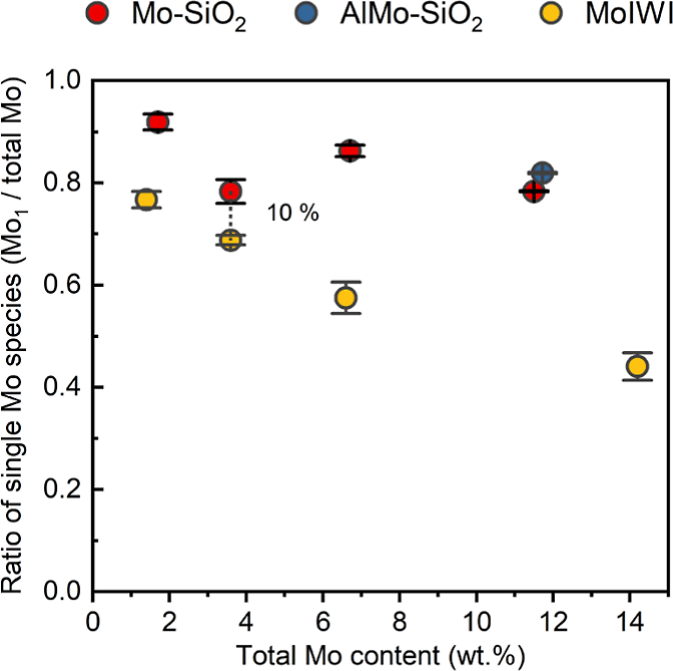
Ratio of single Mo clusters detected by ToF-SIMS for Mo–SiO_2_ and MoIWI. Error bars are the standard deviation of each data set (3 analyses per sample).

The proportion of single Mo clusters for Mo–SiO_2_-based microspheres is summarized in Figure 3, and is compared with MoIWI (Figures S37 and S38). The ratio of single Mo species stayed relatively constant over the range of loadings explored at values of >80% (Figure 3). ToF-SIMS involves collisions between emitted ions and species on the catalyst surface. As a result of these collisions, large surface species such as poly molybdate or molybdenum oxide clusters can break down, forming fragments that contain only one Mo atom. This breakdown can increase the ratio of single Mo species to total Mo species and further enhance the observed extent of Mo dispersion. To accurately characterize the catalyst surface composition, the edge energies estimated from in situ diffuse reflectance ultraviolet–visible spectroscopy (DR UV–vis) were incorporated for all microspheres. The Mo–SiO_2_ microspheres exhibited edge energies above 4.00 eV (Figure S33), consistent with ToF-SIMS results, indicating high surface Mo dispersion across all Mo–SiO_2_ microspheres.^49^ In contrast, the ratio of single Mo species on MoIWI decreased linearly when the loading increased from 1.4 to 14.2 wt % (Figures 3, S37, and S38). In fact, the characterization of MoIWI (Table S5 and Figure S35) showed that the oligomerized MoO_*x*_ species started to appear above Mo loadings of 3.6 wt % and that MoO_3_ crystals were detected above loadings of 6 wt %. Comparing the Mo–SiO_2_-based microspheres with the MoIWI catalysts, the ratio of single Mo species in Mo–SiO_2_ was systematically and significantly higher than that in the corresponding MoIWI catalysts with a similar total Mo content (Figure 3). Given that the dispersion of single Mo species can play a crucial role in the catalytic performance of Mo-based catalysts,^12,18,22^ the remarkably high ratio of highly dispersed MoO_*x*_ species on all Mo–SiO_2_ and Al11Mo–SiO_2_ microspheres highlights the advantage of the presented NHSG synthesis method.

**Figure 4:**
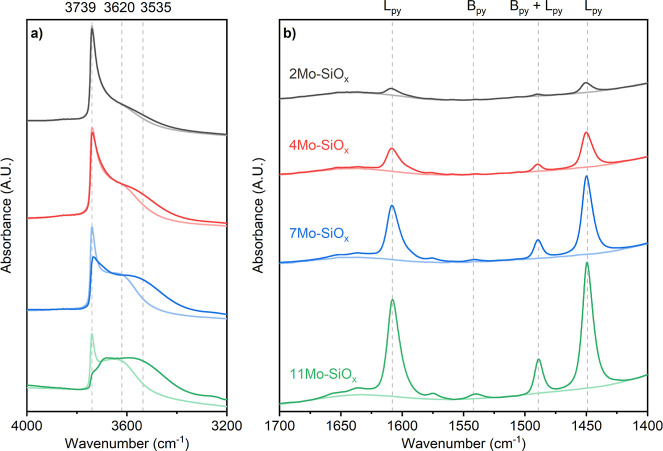
In situ FTIR spectra of prepared catalysts before (light color) and after (dark color) pyridine adsorption (a) in the region 3200–4000 cm^–1^ and (b) in the region 1400–1700 cm^–1^.

The surface acidity of the Mo–SiO_2_ and Al-doped Mo–SiO_2_ microspheres was studied by pyridine adsorption. The FTIR spectra for pristine calcined sample pellets and sample pellets exposed to pyridine are summarized in Figures 4 and S39, where the regions of adsorbed pyridine (1400–1700 cm^–1^) and silanol groups (3200–4000 cm^–1^) are shown. In Figure 4a, the intense sharp band at 3739 cm^–1^ is assigned to isolated or unperturbed Si–OH groups,^42^ and the broad band centered at 3620 cm^–1^ is attributed to perturbed Si–OH groups (e.g., Si–(H)O···Mo(=O)_2_ or Si–O–H···O=Mo=O).^15,52^ Increasing the Mo content resulted in lower signals for isolated Si–OH and stronger signals for perturbed Si–OH. This trend is consistent with the band assignments as the increasing Mo content perturbs more Si–OH on the catalyst. After the surface was saturated with pyridine, an additional decrease in the perturbed Si–OH group band was observed, indicating the existence of H-bonded pyridine on the surface of the catalyst. The growth of the band at 3535 cm^–1^ suggests that more Si–OH groups are perturbed by the adsorption of pyridine. In Figure 4b, the bands at 1449 and 1608 cm^–1^ are attributed to pyridine adsorbed on Lewis acidic Mo centers (L_py_).^53–55^ The band at 1542 cm^–1^ is assigned to pyridine adsorbed on Brønsted acid sites (B_py_).^56^ The band at 1489 cm^–1^ is due to a combination of pyridine adsorbed on Lewis acid and Brønsted acid sites (B_py_ + L_py_). Lewis acid sites are present in all of the catalysts (Figure 4). It has been reported that the Lewis acidic sites are attributed to dioxo (Si–O)_2_Mo(=O)_2_ species in a coordinative unsaturated tetrahedral geometry.^52^ The intensity of the L_Py_ bands (1449 cm^–1^) increased with increasing Mo content. This is likely due to a larger number of dioxo (Si–O)_2_Mo(=O)_2_ species. In contrast to the Lewis acid sites, the amount of Brønsted acid sites was negligible for 2Mo–SiO_2_ and 4Mo–SiO_2_, but it started to increase in 7Mo–SiO_2_. The appearance of measurable Brønsted acidity is likely a consequence of the interaction between silanol groups and adjacent surface species under the formation of pseudobridging Si–O(H)–Mo(=O)_2_ species.^57^

**Figure 5:**
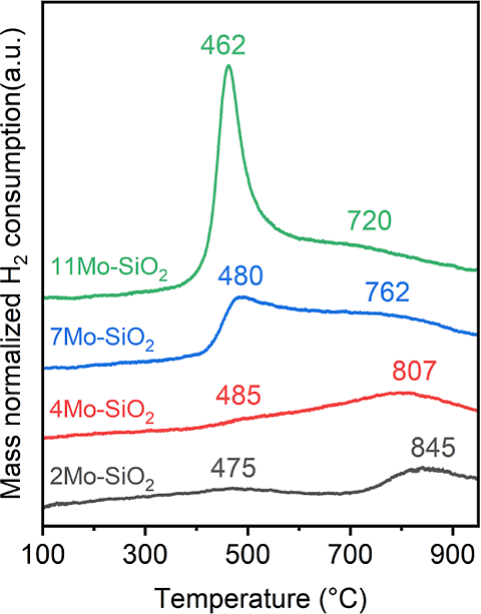
Temperature-programmed reduction (H_2_-TPR) of the Mo–SiO_2_ microspheres. Conditions: 70–80 mg samples measured with a ramping rate of 10 °C min^–1^ in 10% H_2_ in Ar.

H_2_-TPR under 10% H_2_ balanced in Ar was performed to investigate the reducibility of Mo–SiO_2_ microspheres (Figure 5). Two major peaks appeared in the H_2_-TPR profile, suggesting two reduction processes on all Mo–SiO_2_ microspheres. Upon increasing total Mo-content, the intensity of the peak located at low temperatures (ca. 475 °C) increased drastically with negligible peak shift or broadening. In contrast, the center of peaks located at high temperatures (720–845 °C) shifted to low temperatures with progressive broadening, indicating that further reduction of Mo (IV) was facilitated by increasing the Mo content. The absence of obvious H_2_ consumption near 900 °C indicates no MoO_3_ crystalline structures formed on all Mo–SiO_2_ microspheres,^58^ consistent with the Raman and edge energy forms of in situ UV–vis spectra. Previous reports using in situ Raman and X-ray adsorption showed a correlation between the reducibility of surface Mo oxides and their structures, attributing the decrease in temperatures for H_2_ consumption of MoO_*x*_/SiO_2_ to the increase in the internal strain of surface dispersed MoO_*x*_.^59^ Thus, our data suggest enhanced reducibility and thus possibly increased internal strain of MoO_*x*_ species as the total Mo content rises.

### 3.2 Propylene Metathesis Studies

**Figure 6:**
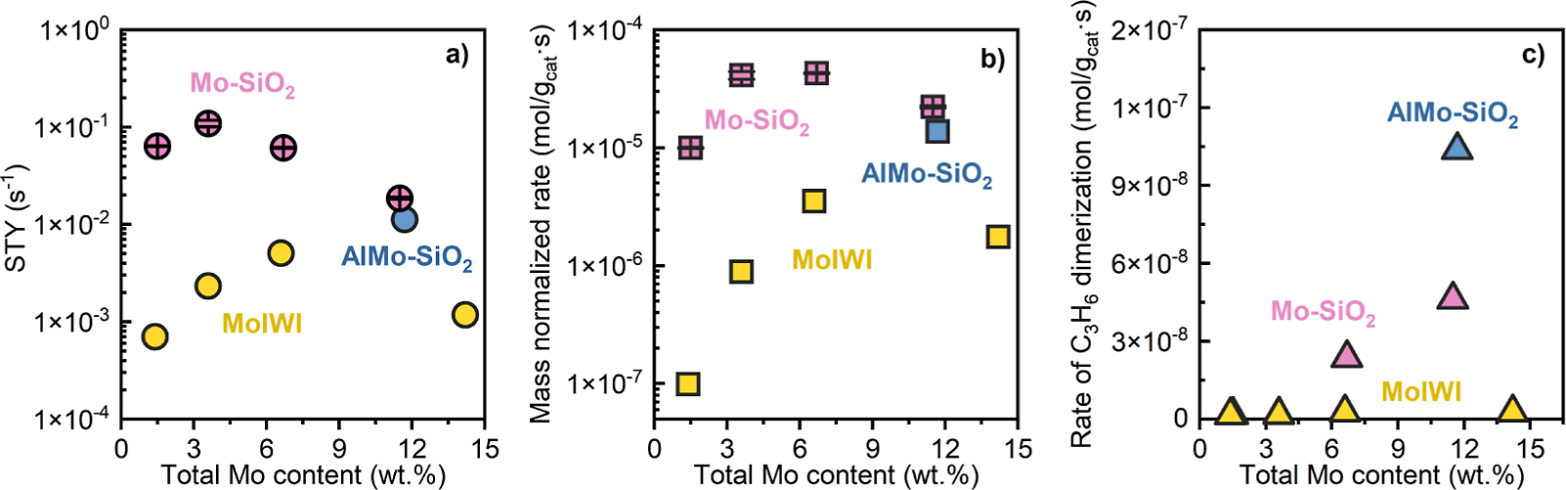
Reactivity of Mo–SiO_2_, Al11Mo–SiO_2_, and MoIWI catalysts, (a) STY of propylene metathesis, (b) rate of propylene metathesis, and (c) rate of propylene dimerization. Conditions: 10–50 mg of catalyst, 50% propylene in a helium balance, and 50 mL min^–1^ total flow rate under atmospheric pressure at 200 °C.

The reactivity of propylene metathesis on prepared Mo–SiO_2_ microspheres was studied and compared to that of MoIWI.^22,23^ The average steady-state Mo content-normalized STY and the catalyst mass-normalized rates of propylene metathesis obtained from multiple beds of each Mo–SiO_2_ are summarized as a function of total Mo content (wt %) in Figure 6. Table S7 summarizes the rate, conversion, and contact times of each catalyst bed. Control experiments show that the reported steady-state rates are acquired under differential conditions (Figure S40), and theoretical estimations show all reported rates are free of mass and heat transfer limitations (Section S10). At steady state, metathesis products—ethylene, *cis*-2-butene, and *trans*-2-butene (molar ratio 2:1:1)—were observed with a total molar selectivity of more than 99%. Throughout the time on stream tested (up to 55 h on 4Mo–SiO_2_), we observed no significant deactivation (Figures S41 and S42). No obvious changes in the reactivity of the 4Mo–SiO_2_ were observed upon two sequential regenerations (Figure S42). In addition to olefin metathesis, previous literature has reported side reactions, mainly thermal cracking and olefin isomerization, caused by the Brønsted acidity of the catalysts.^60,61^ A small quantity of C6-olefins (Figure 6c), mainly methyl-pentene isomers with a total molar selectivity of less than 1%, were observed for microspheres with Mo content higher than 4 wt %, consistent with the FTIR data showing pyridinium formation on the Brønsted acid sites on this catalyst (Figure 4). The detected products indicate two concurrent reaction pathways: olefin metathesis and Brønsted acid site-catalyzed olefin dimerization, with a strong predominance of the former.^55^

Both the STY and the rate of propylene metathesis over MoIWI catalysts increased with the Mo content and reached a maximum when the 7MoIWI sample was used (Figure 6). The observed trend in the propylene metathesis rate over MoIWI catalysts was similar to previous studies,^59^ in which an exponential growth in propylene metathesis rate was observed over catalysts with no MoO_3_ crystallites—an effect attributed to an increasingly strained MoO_*x*_ surface. Raman spectra of 7MoIWI and 14MoIWI (Figure S35) indicate the presence of MoO_3_ crystals on the catalyst surface. Previous studies have proposed that oligomerized MoO_*x*_ and crystalline MoO_3_ are inactive for olefin metathesis.^20,21^ With the presence of MoO_3_ on 7MoIWI, the increase in the rate of propylene metathesis from 4MoIWI to 7MoIWI is attributed to the increased strain effect apparent in the highly dispersed MoO_*x*_ on 7MoIWI, outweighing the smaller amount of MoO_3_ on 7MoIWI. Finally, the oligomerized MoO_*x*_ and crystalline MoO_3_, which make up more than half of the total Mo content (Figure S37), dominate the 14MoIWI surface, resulting in a decrease in the overall metathesis reactivity of the catalyst.

Steady-state propylene metathesis rates over the Mo–SiO_2_ microspheres were nearly 2 orders of magnitude larger than rates over the MoIWI catalyst with the lowest Mo content (<4 wt % Mo) at 9.96 × 10^–6^ mol g_cat_^–1^ s^–1^ on 2Mo–SiO_2_. The rate increased with total Mo content, reaching a maximum of 4.11 × 10^–5^ mol g_cat_^–1^ s^–1^ for 4Mo–SiO_2_ and then decreasing to 2.22 × 10^–5^ mol g_cat_^–1^ s^–1^ for 11Mo–SiO_2_. For comparison, the highest rate observed with catalyst prepared by the impregnation method reached 3.51 × 10^–6^ mol g_cat_^–1^ s^–1^ for 6.6% MoIWI. Our recent study of MoO_*x*_/SiO_2_ with a 1.5 wt % Mo loading synthesized through the surface organometallic method (MoSOMC) showed a propylene rate of 1.64 × 10^–6^ mol g_cat_^–1^ s^–1^ at similar reaction conditions.^62^ All of these comparisons demonstrate the excellent catalytic performance of Mo–SiO_2_ microspheres in heterogeneous olefin metathesis.

For the Mo–SiO_2_ microspheres, the site reactivity of propylene metathesis initially increased as total Mo content increased from 2 to 4 wt %. Similar to the MoIWI catalysts, we attribute the major factor governing the initial increase to the internal strain within the surface-dispersed Mo oxides.^59^ We also observed a slight decrease in metathesis rate when using microsphere catalysts containing more than 4 wt % Mo. The addition of 1 wt % Al into 11Mo–SiO_2_ (Al11Mo–SiO_2_) resulted in an increase in the quantity of Brønsted acid sites, as evidenced by pyridine adsorption (Figure S39), and the surface molybdenum content, as determined by XPS (Table S2), while preserving a high level of molybdenum dispersion (Figures S33 and S37). However, the catalytic activity of Al11Mo–SiO_2_ in propylene metathesis was inferior to that of 11Mo–SiO_2_ (Figure 6). Since the dispersion of MoO_*x*_ on all microspheres remains relatively constant (Figure 3 and Table 2), the decrease in metathesis rate from the increasing oligomerization of surface MoO_*x*_, which is associated with increasing Mo loading and usually reported as a major reason for losing metathesis activity on high Mo loading catalysts,^20,21^ should be minor.

**Figure 7:**
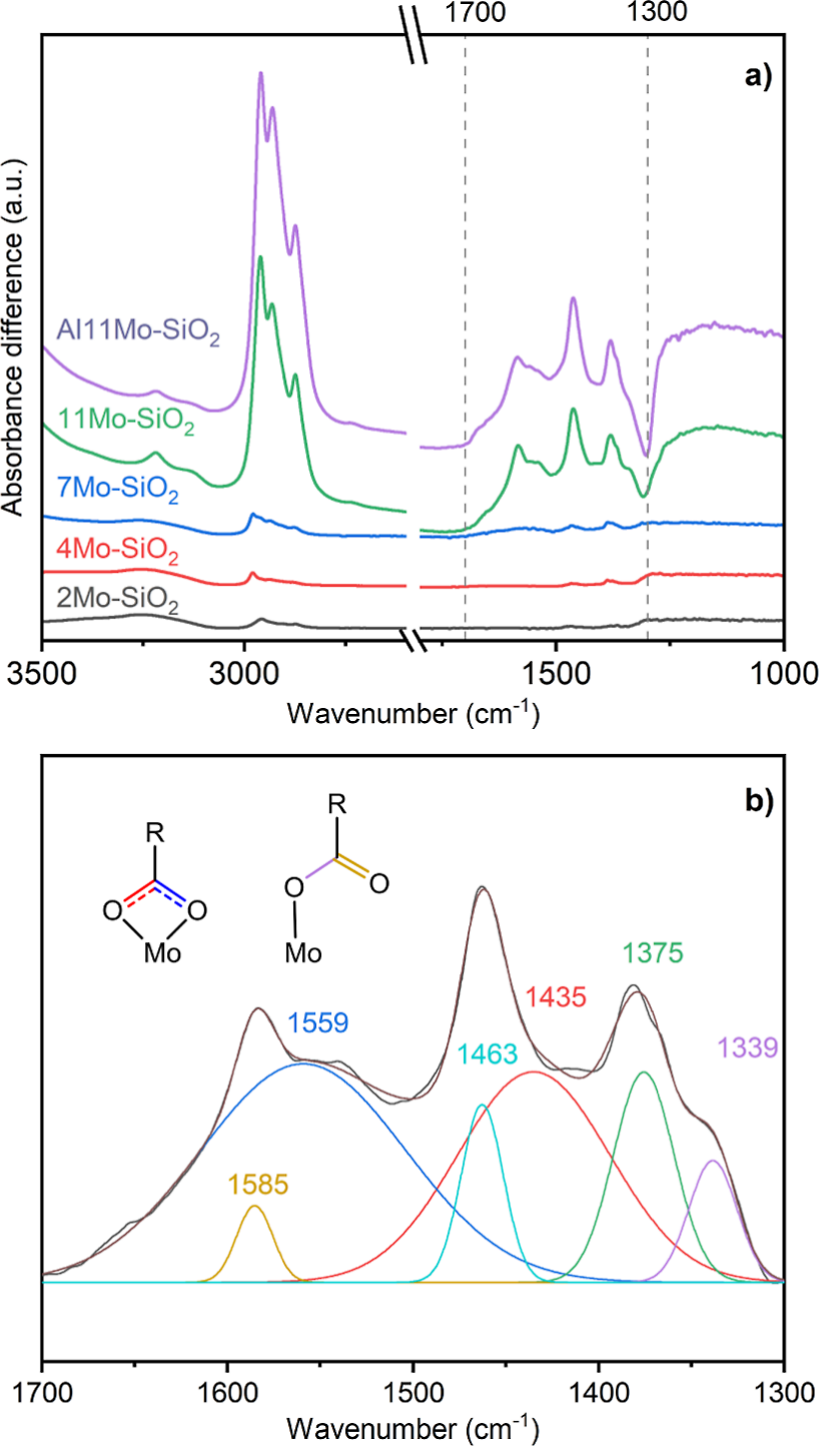
(a) In situ transmissive FT-IR spectra for Mo–SiO_2_ and Al11Mo–SiO_2_ microspheres post adsorption of 20% propylene at 200 °C. Vertical offsets were applied to separate each spectrum. (b) The peak deconvolution for the spectrum on 11Mo–SiO_2_ ranges from 1300 to 1700 cm^–1^.

We then investigated why the metathesis reactivity on Mo–SiO_2_ decreased with Mo content higher than 4 wt % by screening the catalyst surfaces after adsorption of propylene at reaction temperature (200 °C). After the adsorption of propylene, the in situ FTIR spectra (Figure 7a) for 11Mo–SiO_2_ and Al11Mo–SiO_2_ showed intense absorption bands in the regions of 1300 to 1700 cm^–1^ and 2800 to 3300 cm^–1^. Peak deconvolution (Figure 7b) revealed 6 peaks from the 1300 to 1700 cm^–1^ region, where the two most intense peaks located at 1559 and 1435 cm^–1^ are most likely ν_as_ (O–C–O) and ν_s_ (O–C–O) associated with surface carboxylate species.^63^ The difference between their peak centers (Δν) is 124 cm^–1^, suggesting an adsorbed chelating bidentate carboxylate structure.^64^ The peaks at 1585 and 1339 cm^–1^ could be due to ν(C=O) and ν(C–O) in surface carboxylic species with a Δν of 246 cm^–1^, which is representative of monodentate carboxylates.^64^ The peaks at 1463 and 1375 cm^–1^ could be due to δ (C–H), peaks located from 2800 to 3000 cm^–1^ should be due to ν(C–H) for sp^3^ C–H bonds, and peaks at 3124 and 3217 cm^–1^ are likely associated with ν(C–H) for sp^2^ C–H bonds.^63^

The surface carboxylate species on 7Mo–SiO_2_, 11Mo–SiO_2_, and Al11Mo–SiO_2_ can be generated from the reaction between adsorbed propylene and lattice oxygen in MoO_*x*_. An increasing amount of these surface carboxylate species is consistent with the enhanced reducibility of the Mo–SiO_2_ microspheres with an increasing total Mo content (H_2_-TPR, Figure 5). Once formed, the surface carboxylates are generally stable under our reaction conditions,^65,66^ hindering the access of propylene to the Mo sites and thereby inhibiting propylene self-metathesis. We also observed that absorption bands assigned to ν(C–H) and δ (C–H) for sp^3^ C–H appeared on 2Mo–SiO_2_ and 4Mo–SiO_2_, without any signals for carboxylates, suggesting the presence of other surface species. Since absorption bands associated with sp^3^ C–H bonds in surface carboxylic species are typically less intense than the corresponding peaks due to ν_as_ (O–C–O) and ν_s_ (O–C–O),^67,68^ there should also exist surface species that contribute to the appreciable ν(C–H) bands on 7Mo–SiO_2_ and 11Mo–SiO_2_. Indeed, we have previously shown the formation of surface alkoxides post propylene adsorption on the surface of MoO_*x*_/SiO_2_ and WO_*x*_/SiO_2_ catalysts.^62^ Therefore, one plausible source for the peaks associated with the sp^3^ C–H bond on Mo–SiO_2_ microspheres could be alkoxide formed by the adsorption of propylene onto a Brønsted acid site. Since C6 olefins were observed as products from propylene dimerization on 7Mo–SiO_2_, 11Mo–SiO_2_, and Al11Mo–SiO_2_ (Figure 6c), the weak sp^2^ ν(C–H) (3124 and 3217 cm^–1^) on the spectrum of 11Mo–SiO_2_ and Al11Mo–SiO_2_ could be attributed to olefins generated through propylene dimerization or further polymerization catalyzed by the Brønsted acid site.^60,61^ Therefore, we attribute the decreasing trend in the metathesis reactivity from 4Mo–SiO_2_ to Al11Mo–SiO_2_ to surface poisoning by surface carboxylic species and surface olefins.

Propylene dimerization was observed on Mo–SiO_2_ microspheres with a Mo content of 6.7 wt % or higher (Figure 6c). The increasing trend in the rate of propylene dimerization from 7Mo–SiO_2_ to Al11Mo–SiO_2_ is consistent with the observed trend in pyridine adsorption (Figures 4 and S39), where the signatures of adsorbed pyridine on Brønsted acid sites were initially observed on 7Mo–SiO_2_ and then increased with higher Mo content and the addition of Al. Contrarily, the propylene metathesis rate displayed an inverse pattern, starting to decline with a Mo loading above 6.7 wt % and with the incorporation of Al.

Besides site blocking caused by surface carboxylic species (as shown in Figure 7), another potential explanation for the diminished metathesis reactivity on 7Mo–SiO_2_, 11Mo–SiO_2_, and Al11Mo–SiO_2_ could be propylene polymerization occupying Brønsted acid sites. These sites are essential for the 1,2-proton shift mechanism to transition Mo oxides to Mo alkylidene.^14,62,69^ The influence of Brønsted acid sites on steady-state metathesis reactivity can be rationalized by a recently proposed site renewal and decay cycle (Scheme S1) that coexists with propylene metathesis on WO_*x*_/SiO_2_ and MoO_*x*_/SiO_2_ catalysts.^62^ The proposed cycle emphasizes the importance of the Brønsted acidity of the corresponding proton donor—perturbed Si–OH (Section S12). In this manner, it is reasonable to foresee that the propylene dimerization process (Figure 6c), catalyzed by Brønsted acid sites, could compete with the formation of metal alkylidenes on perturbed Brønsted acidic Si–OH. Consequently, this competition hinders the participation of perturbed Si–OH in the proton transfer process during the site renewal steps (Scheme S1), leading to a decreased surface coverage of metal alkylidenes. Ultimately, this diminished coverage could potentially result in a low steady-state rate for propylene metathesis. Notably, the propylene dimerization rate is at least 2 orders of magnitude slower than the metathesis rate, suggesting that the negative impact of propylene dimerization on propylene metathesis would be minor.

Small clusters of MoO_*x*_, including dimers and trimers, could be present on Mo–SiO_2_ microspheres with a high Mo content. These clusters are less reactive for olefin metathesis and can thus contribute to lower catalyst mass/Mo content normalized reactivities of catalysts. Detecting the presence of these small MoO_*x*_ clusters poses a challenge as they could be difficult to observe through in situ UV–vis or SIMS techniques. Ongoing efforts are being made to identify the presence of small MoO_*x*_ clusters utilizing solid-state ^95^Mo NMR spectroscopy.

## 4 Conclusions

In this work, we disclose the preparation of new molybdenum silicate microspheres that exhibit outstanding catalytic activity for olefin metathesis. The synthesis method for molybdenum-silicate spheres involves microwave-assisted preparation of the Mo-Bpdc hybrid organic–inorganic precursor and its subsequent condensation with (3-aminopropyl)triethoxysilane under nonaqueous conditions. This synthesis method generated monodisperse hybrid molybdenum silicate motifs with tunable Mo loadings. The calcined molybdenum silicate microspheres exhibited an amorphous and porous character, with a uniform distribution of highly dispersed molybdenum species. The superior dispersion of molybdenum oxide species in the microspheres was confirmed by ToF-SIMS and in situ UV–vis.

The molybdenum species are represented mostly by dioxo (Si–O)_2_Mo(=O)_2_ species in a coordinative unsaturated tetrahedral geometry that showed remarkable catalytic activity for the propylene metathesis reaction compared to traditional catalysts prepared with IWI under identical reaction conditions. More specifically, Mo–SiO_2_ microspheres with low Mo content (1.5–3.6 wt %) exhibited steady-state rates nearly 2 orders of magnitude higher than those for the MoIWI catalysts with similar Mo loading. We attributed the high activity on Mo–SiO_2_ microspheres with a 1.5–3.6 wt % Mo content to their high dispersion of molybdenum oxide (pre)active sites and appropriate Brønsted acidity that would not catalyze the side reaction.

## Supporting Information Available

Study of the Mo-Bpdc-Si solid precursors; FTIR spectroscopy and powder X-ray diffraction of prepared materials; solid-state NMR characterization; SEM–EDX and XPS analyses of the catalysts; nitrogen adsorption–desorption isotherms; DR UV–vis spectra, *E*_g_ values, and Raman spectra; ToF SIMS analysis results; in situ FTIR spectra for pyridine adsorption on an Al11Mo–SiO_2_ sample; reactivity results; propylene metathesis rate of Mo–SiO_2_ microspheres as a function of time on stream; site renewal and decay cycle together with the Chauvin cycle; and influence of product co-feeding on the steady state rates (PDF)
