# Supplementary material for: Propylene Metathesis over Molybdenum Silicate Microspheres with Dispersed Active Sites
Source: ACS Catal. 2023 Sep 20;13(19):12970–82. doi: 10.1021/acscatal.3c02045 (PMC10563125; doi:10.1021/acscatal.3c02045)
Supplement: Supplementary file 1 — cs3c02045_si_001.pdf [file cs3c02045_si_001.pdf]

Supporting Information

Propylene Metathesis over Molybdenum Silicate Microspheres with Dispersed Active Sites

David Skoda [1§*], Ran Zhu [2§], Barbora Hanulikova [1], Ales Styskalik [3], Vit Vykoukal [3,4], Petr Machac [3], Lucie Simonikova [3], Ivo Kuritka [1], Claude Poleunis [5], Damien P. Debecker [5*], Yuriy Román-Leshkov [2*]

[1] Centre of Polymer Systems, Tomas Bata University in Zlin, tr. Tomase Bati 5678, Zlin, CZ-76001, Czech Republic

[2] Department of Chemical Engineering, Massachusetts Institute of Technology (MIT), 77 Massachusetts Avenue, Cambridge, MA 02139, USA.

[3] Department of Chemistry, Faculty of Science, Masaryk University, Kotlarska 2, Brno, CZ-61137, Czech Republic

[4] Central European Institute of Technology, Masaryk University, Kamenice 5, Brno, CZ 62500, Czech Republic

[5] Institute of Condensed Matter and Nanosciences (IMCN), Université catholique de Louvain (UCLouvain), Place Louis Pasteur 1, 1348 Louvain-La-Neuve, Belgium

* dskoda@utb.cz

* yroman@mit.edu

* damien.debecker@uclouvain.be

Table of contents

S1. Study of the Mo-Bpdc-Si solid precursors 2

S2. FTIR spectroscopy and powder X-Ray diffraction of prepared materials 3

S3. Solid-state NMR characterization 6

S4. SEM-EDX elemental analysis 8

S5. XPS analysis of the catalysts 15

S6. Nitrogen adsorption-desorption isotherms 22

S7. DRUV-Vis E_g values and Raman spectroscopy 23

S8. ToF SIMS results 26

S9. In-situ FTIR for pyridine adsorption on Al11Mo-SiO_2 28

S10. Supplementary of the reactivity results 29

S11. Propylene metathesis rate of Mo-SiO_2 microspheres as a function of time on stream 31

S12. The site renewal and decay cycle 33

S13. Influence of product co-feeding on the steady state rates. 34

S14. References 35

S1. Study of the Mo-Bpdc-Si solid precursors

Table S1. The precursor amounts used for the synthesis of the catalysts

Sample	m_{Mo} [g]	n_{Mo} [mmol]	m_{Al} [g]	n_{Al} [mmol]	m H_2BPDC [g]	n H_2BPDC [mmol]	m_{APTES} [g]	n_{APTES} [mmol]	Yield [g]
11Mo-SiO_2	0.1644	0.503			0.2436	1.006	1.145	5.172	0.546
7Mo-SiO_2	0.0806	0.245			0.2434	1.005	1.123	5.074	0.582
4Mo-SiO_2	0.0403	0.124			0.2423	1.000	1.122	5.070	0.554
2Mo-SiO_2	0.0212	0.065			0.2428	1.002	1.131	5.109	0.452
Al11Mo-SiO_2	0.1640	0.503	0.3249	1.002	0.2425	1.001	1.235	5.579	0.723

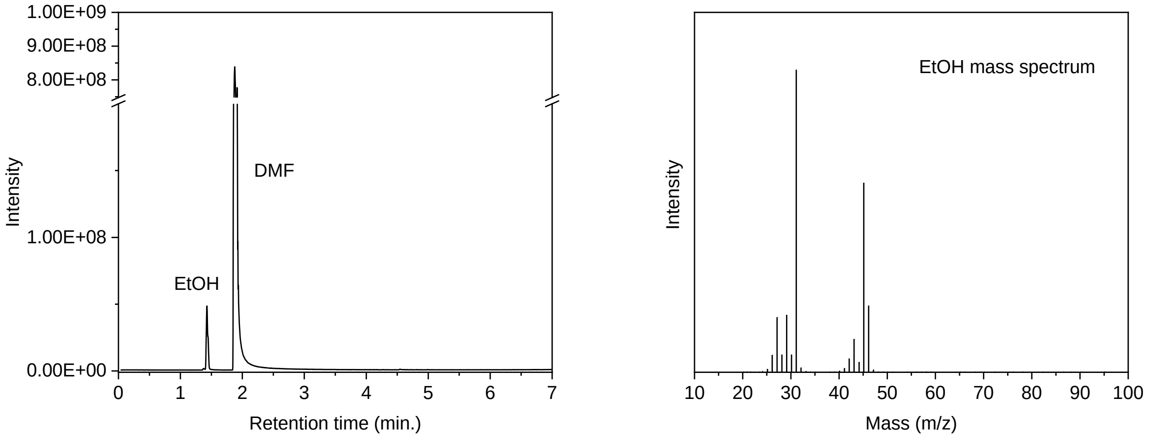

Figure S1. *GC-MS chromatogram of 11Mo-SiO_2 reaction byproduct (left). MS spectrum of EtOH byproduct (r.t. 1.47 min.)*

TGA analysis was performed to investigate the thermal behavior of Mo-Bpdc-Si solid precursors. The thermogravimetric curves recorded in the air atmosphere of all samples are given in Figure S2. As demonstrated, they exhibited almost the same shape with comparable mass losses assigned to oxidation and decomposition of residual functional groups (3-aminopropyl or ethoxy) and Bpdc fragments.[1]

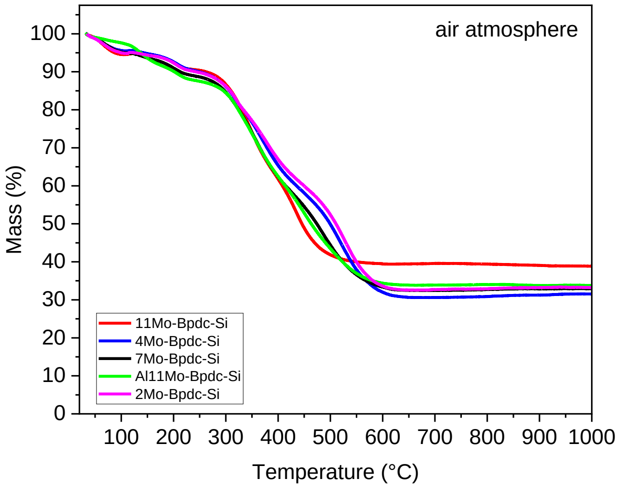

Figure S2. *Thermogravimetric curves of Mo-Bpdc-Si solid precursors.*

S2. FTIR spectroscopy and powder X-Ray diffraction of prepared materials

FTIR spectra of all Mo-Bpdc-Si solid precursors (Figure S3) exhibited an intense absorption band at 1375 cm^{-1} which is attributed to the symmetric stretching modes of carboxylate groups in Bpdc linker.[2] The vibrational bands located in regions 1525 and 1579 cm^{-1} are ascribed to the asymmetric stretching modes of carboxylate groups. The Si–O–Si linkages are characterized by vibrational bands located at 1118, 1033, and 1005 cm^{-1}.[3] A vibrational band with wavenumber 960 cm^{-1} is indicative for Si–OH/Si–O–Mo species.[4] Further, the vibrational band of Mo=O bonds is found at 917 cm^{-1}.[5]

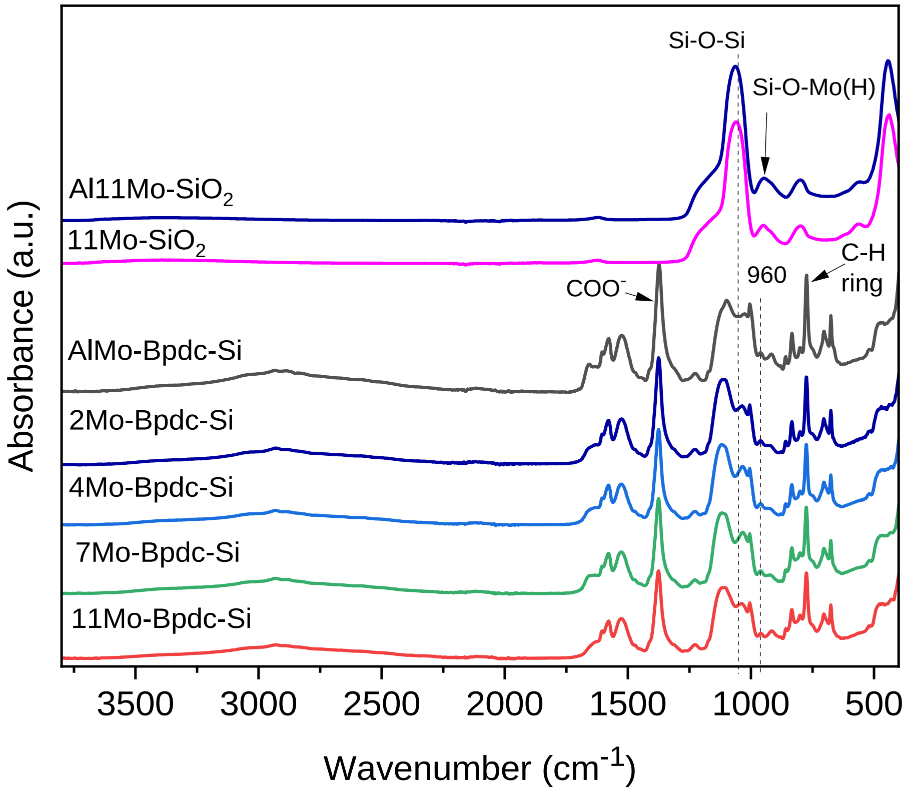

Figure S3. *FTIR spectra of Mo-Bpdc-Si solid precursors (ATR technique).*

FTIR spectra of the microspheres with 11 wt.% Mo content (Figure S3) exhibited intense vibrational bands at 1060 and 440 cm^{-1} indicative of Si–O–Si bonds.[3] As illustrated in Figure S4, the intensity of the vibrational band at 952 cm^{-1} increased with the Mo content. This vibrational band (952 cm^{-1}) could be attributed to the stretching mode of both Si–O-Mo and Si–OH bonds.[4,6,7]

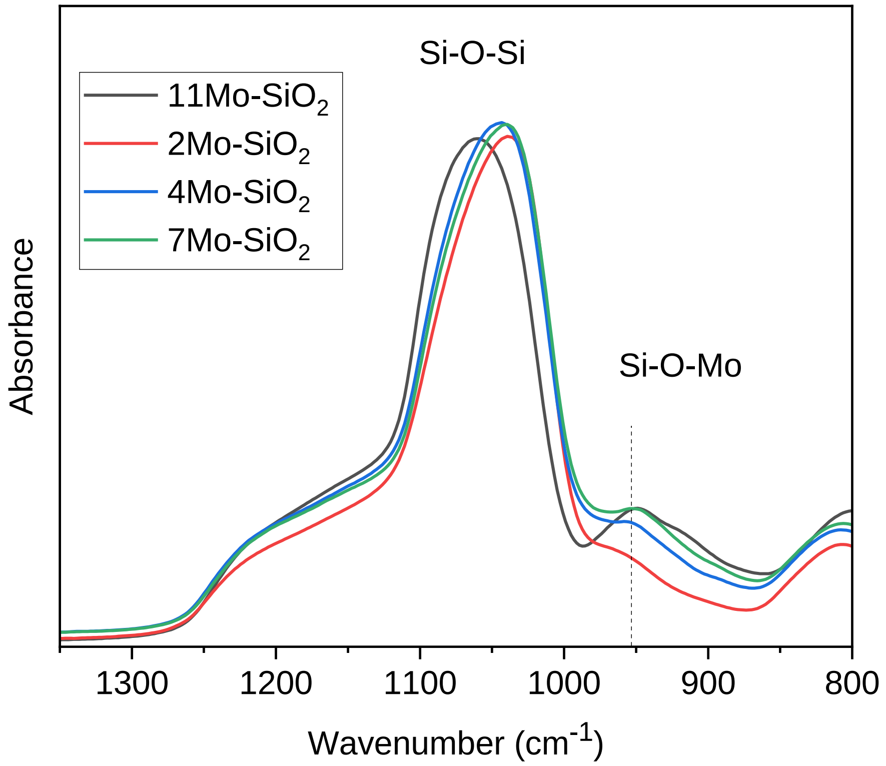

Figure S4. *FTIR spectra of Mo-SiO₂ microspheres (region of Mo–O–Si and Si–O–Si linkages).*

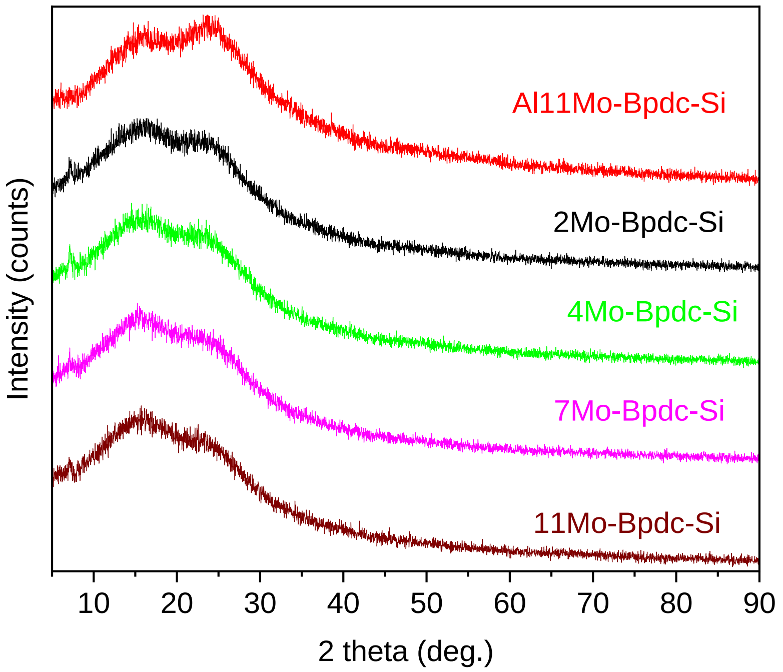

Figure S5. *Powder XRD patterns of Mo-Bpdc-Si solid precursors.*

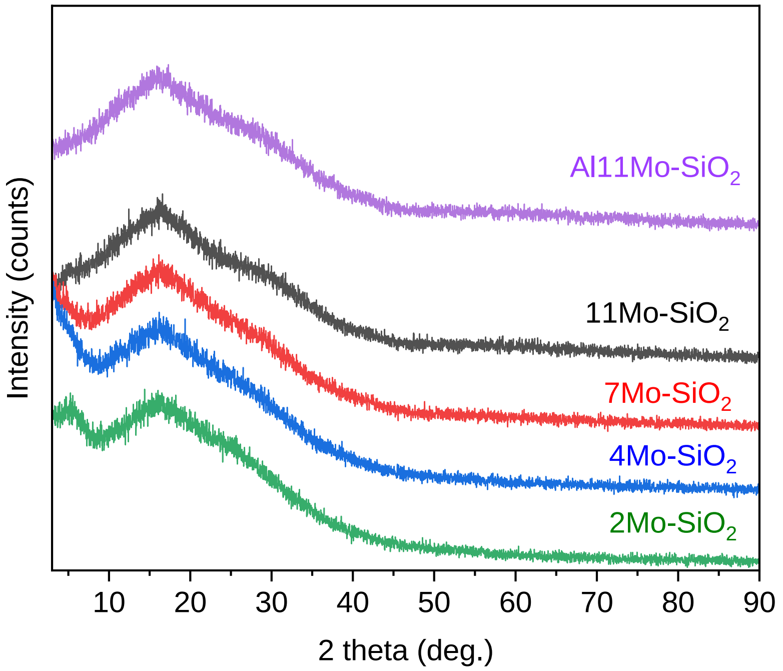

Figure S6*. XRD diffraction patterns of Mo-SiO_2 and AlMo-SiO_2 microspheres.*

S3. Solid-state NMR characterization

^{29}Si CPMAS NMR spectrum of 11Mo-Bpdc-Si sample is shown in Figure S7. The spectrum shows two signals with the chemical shifts –60.0 and –66.6 ppm denoted as T_2 and T_3 type groups, respectively (Figure S7).[8] These groups are represented by L–Si(OSi)$_2$(OR) species for T_2 and L–Si(OSi)$_3$ sites for T_3, where L represents the 3-aminopropyl group and R: Bpdc or ethoxy group observed also in ^{13}C CPTOSS MAS NMR spectrum (Figure S7 on the right).[1] The same signals are observed in the ^{29}Si CPMAS NMR spectrum of the AlMo-Bpdc-Si sample. The results obtained from the ^{29}Si CP MAS NMR spectrum of the Mo-Bpdc-APTES sample confirmed the condensation of APTES.

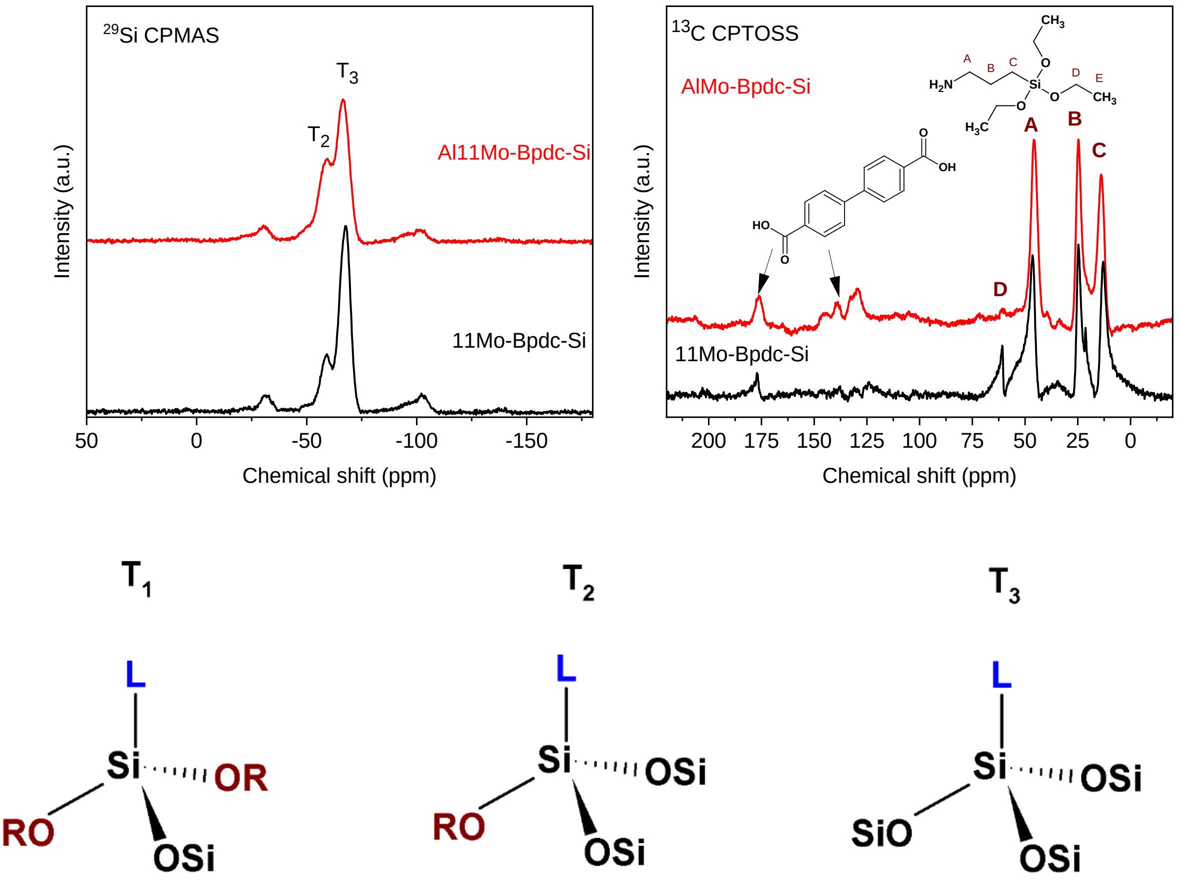

Figure S7. *^{29}Si CPMAS NMR spectra of as-prepared Mo-SiO$_2$ samples.*

^{29}Si CPMAS NMR spectra of molybdenum silicate catalysts exhibited the signals corresponding to Q_n sites in silica-based materials. This observation proved the successful transformation of as-prepared hybrid organosiloxanes to fully inorganic silicates upon calcination, which can also be indicated by the absence of T sites in ^{29}Si CPMAS NMR spectra in Figure S7. As shown in ^{29}Si CPMAS NMR spectra (Figure S8), the most intense signal is centered at –101.1 ppm, which is assigned to Q_3 site (MoO)Si(OSi)$_3$/HO-Si(OSi)$_3$. Besides this signal, there are shoulder peaks with the chemical shifts –110.0 and –91.9 ppm, which are attributed to Q_2 site (MoO)$_2$Si(OSi)$_2$/(HO)$_2$Si(OSi)$_2$ and Q_4 site Si(OSi)$_4$, respectively.[4,8,9]

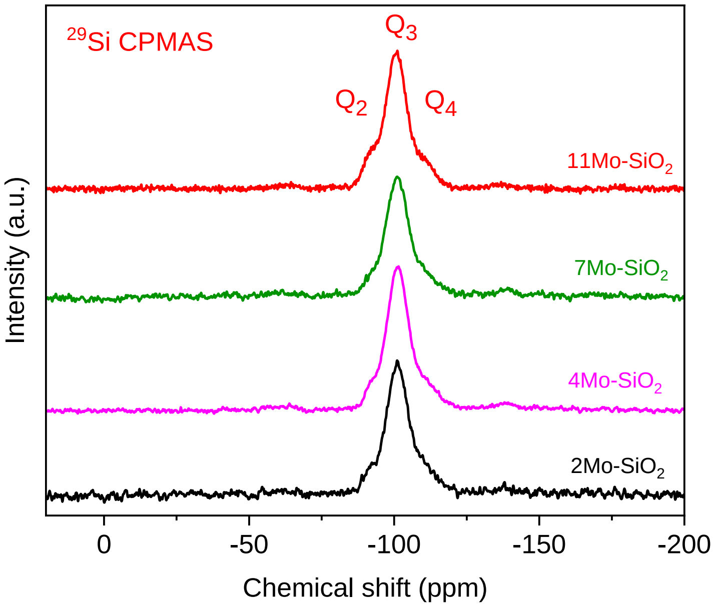

Figure S8. *^{29}Si CPMAS NMR spectra of Mo-SiO_2 microsphere.*

^{29}Si CPMAS NMR spectrum of Al11Mo-SiO_2 catalyst displayed in Figure S9 shows the most intense signal is centered at –101.1 ppm and it is assigned to Q_3 site (MoO)Si$(OSi)_3$/HO-Si$(OSi)_3$. Besides this signal, there are shoulder peaks with the chemical shifts –110.0 and –91.9 ppm, which are attributed to Q_2 site $(MoO)_2Si(OSi)_2/(HO)_2Si(OSi)_3$ and Q_4 site $Si(OSi)_4$, respectively.[4,8,9]

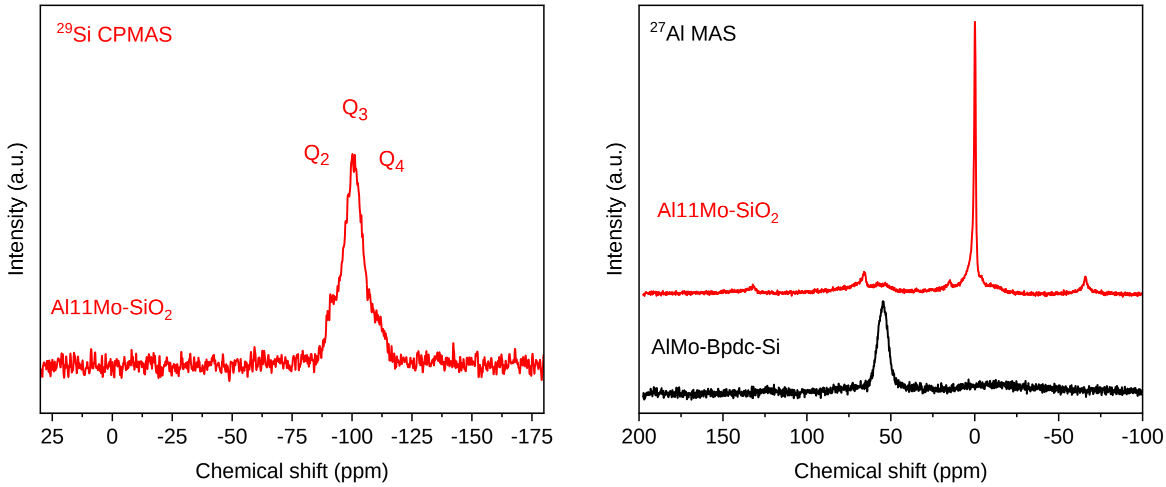

Figure S9. *Left: ^{29}Si CPMAS NMR spectrum of Al11Mo-SiO_2 microsphere. Right: ^{27}Al MAS NMR spectrum of AlMo-SiO_2 silicate sample (solid precursor: black, calcined: red).*

^{27}Al MAS NMR spectrum of Al11Mo-SiO_2 catalyst revealed an intense and sharp signal of six-coordinated aluminum raised at –0.15 ppm.[10] As seen, the signal of four-coordinated aluminum at 53 ppm found in the case of Al11Mo-Bpdc-Si solid precursor has been dramatically decreased (Figure S9 on the right). This observation can be connected to the oxidation of aluminum species in catalyst and thus increase the aluminum coordination number.

S4. SEM-EDX elemental analysis

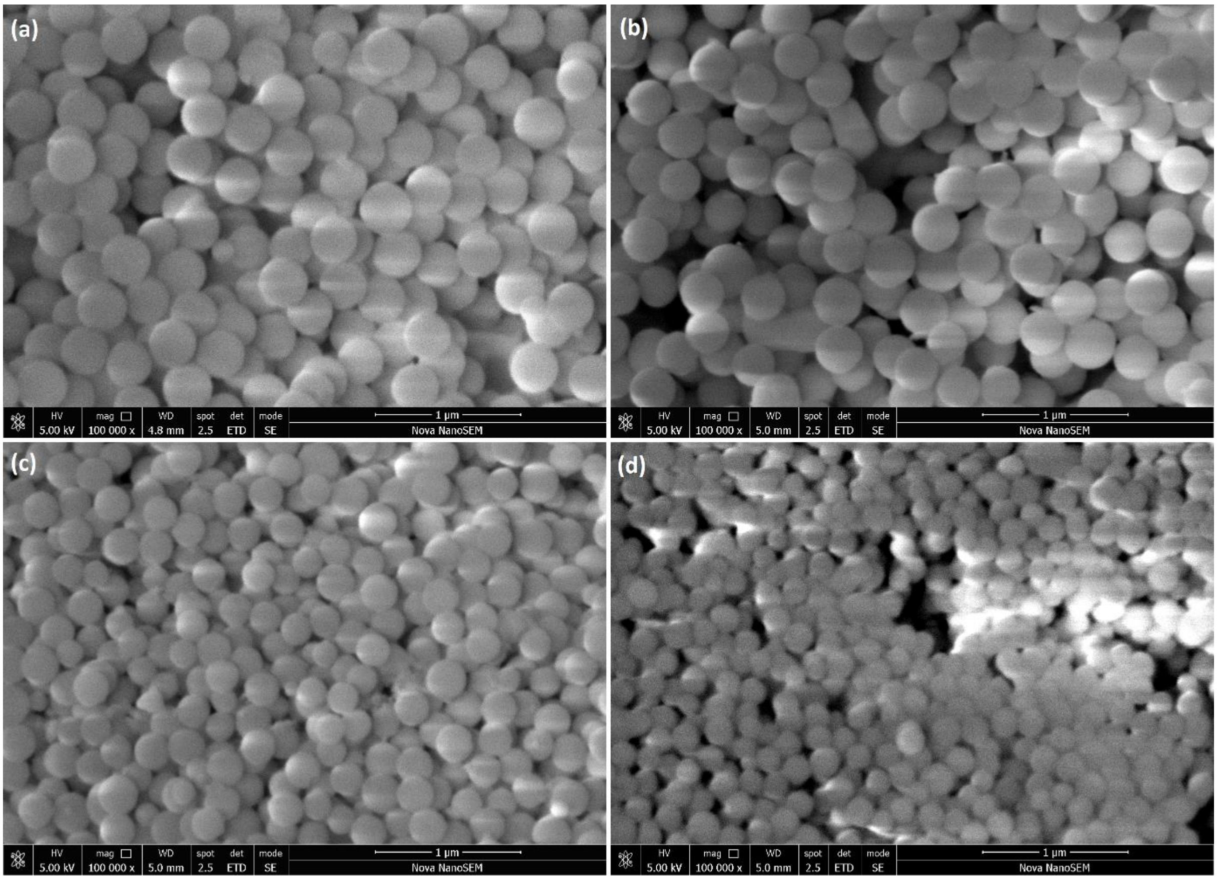

Figure S10. *SEM images of (a) 11Mo-SiO$_2$, (b) 7Mo-SiO$_2$, (c) 4Mo-SiO$_2$ (d) 2Mo-SiO$_2$ catalyst samples.*

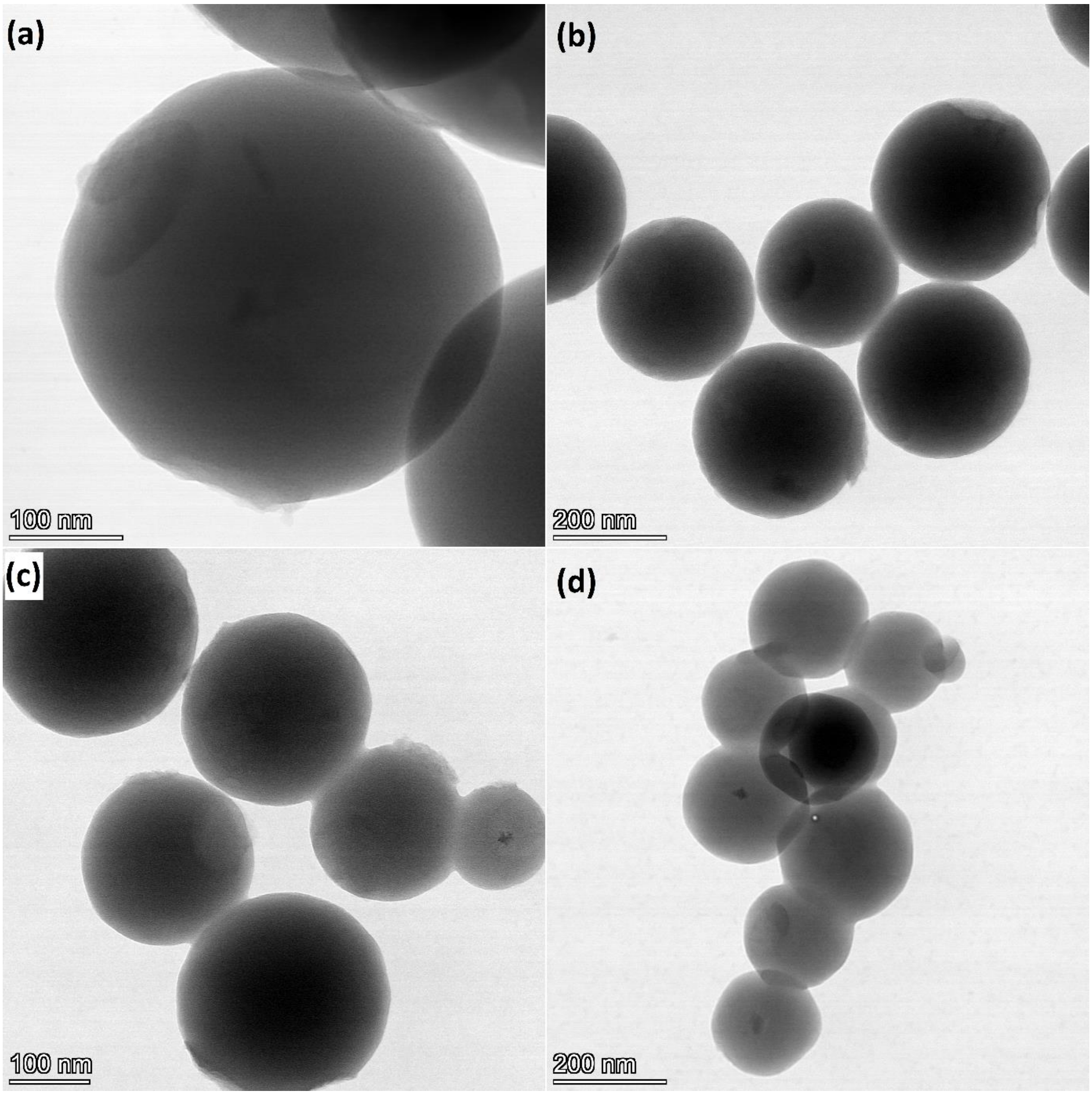

Figure S11. *TEM images of (a) 11Mo-SiO_2, (b) 7Mo-SiO_2, (c) 4Mo-SiO_2 (d) 2Mo-SiO_2 catalyst samples.*

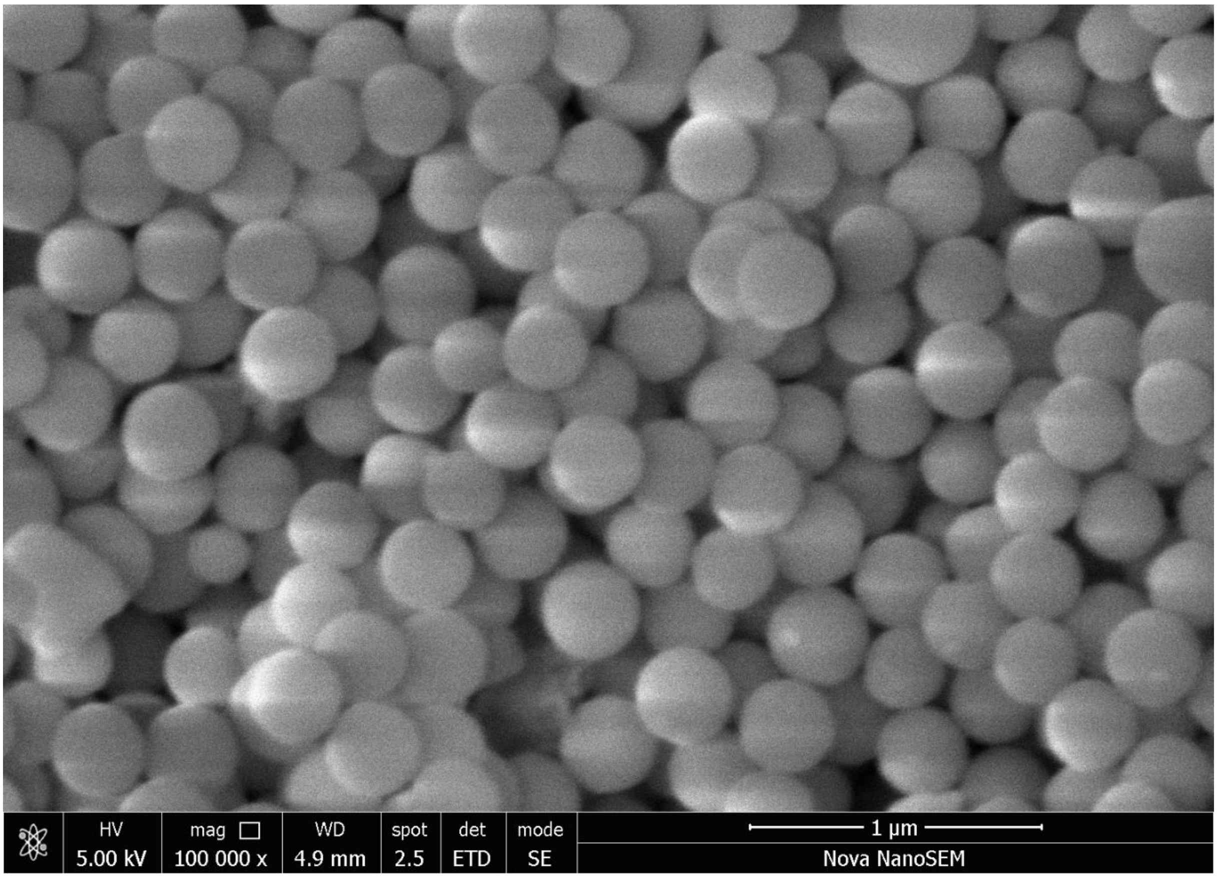

Figure S12. *SEM image of Al11Mo-SiO_2 catalyst sample.*

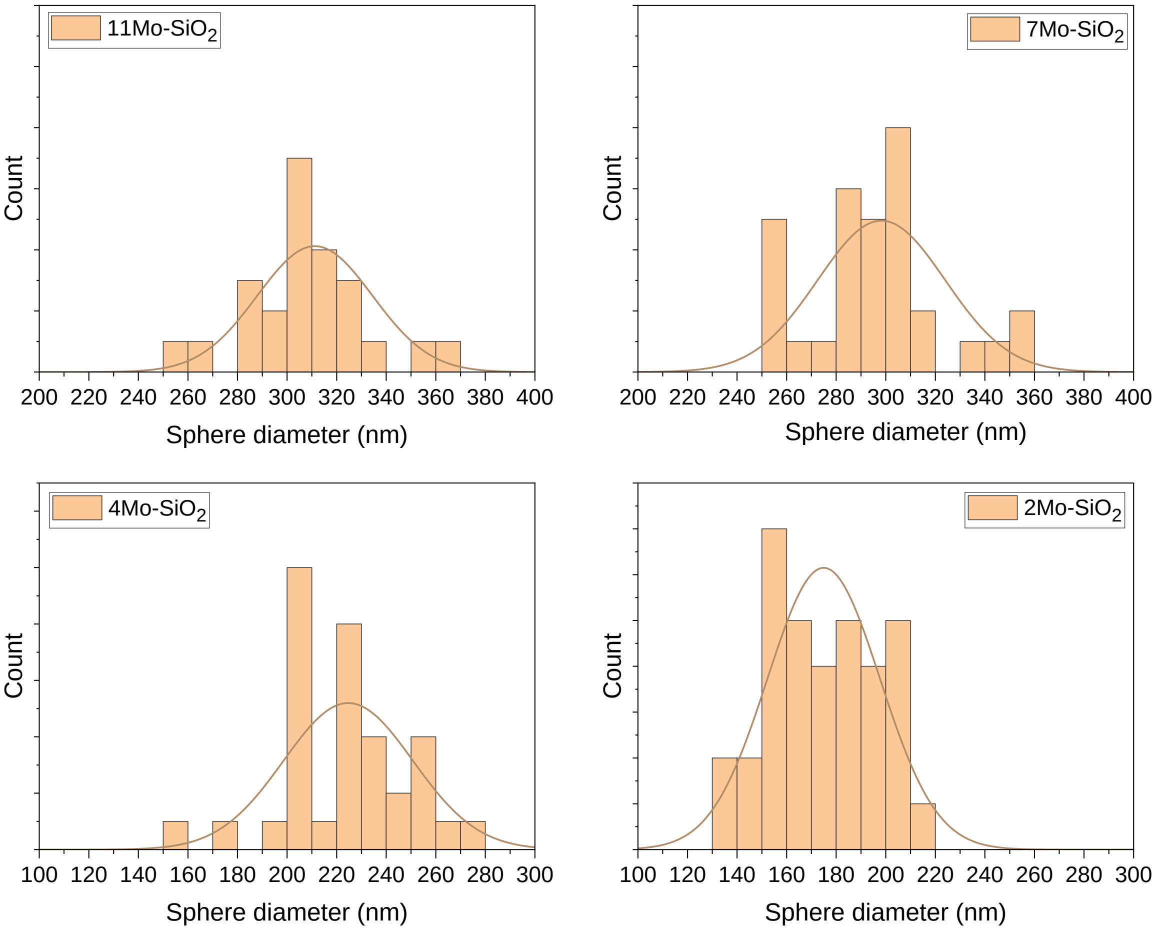

Figure S13. *Mo-SiO$_2$ microsphere diameter histograms with distribution curves. Estimated by electron microscopy images.*

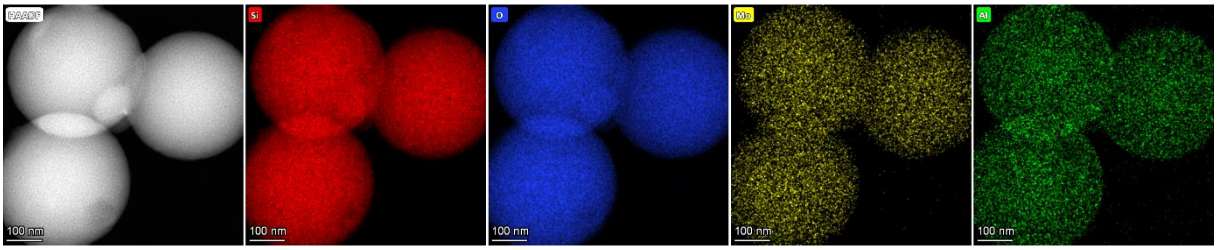

Figure S14. *STEM-EDS elemental maps of Al11Mo-SiO$_2$ catalyst* (red – Si, blue – O, yellow – Mo, green - Al).

Table S2. Elemental contents are determined by SEM-EDX and XPS techniques.

Sample	wt.% EDX					wt.% XPS				
	Si	O	Mo	C	Al	Si	O	Mo	C	Al
11Mo-SiO_2	37.74	47.07	10.49	5.1		42.74	46.6	6.26	4.41	
7Mo-SiO_2	42.24	46.87	6.72	4.18		48.15	43.8	3.5	4.54	
4Mo-SiO_2	43.3	48.16	3.65	4.87		50.13	44.52	2.29	3.3	
2Mo-SiO_2	36.72	55.00	1.59	6.7		49.69	45.15	0.79	3.20	
Al11Mo-SiO_2	37.89	46.37	10.04	4.74	0.96	42.85	42.32	10.51	5.84	2.7

Table S3. Comparison of Si, Mo, and Al elements content determined by ICP-OES.

Sample	ICP [wt%]		
	Si	Mo	Al
11Mo-SiO_2	34.4	11.49	
7Mo-SiO_2	37.91	6.65	
4Mo-SiO_2	39.77	3.62	
2Mo-SiO_2	42.53	1.70	
Al11Mo-SiO_2	31.99	11.72	0.50

The nominal Si/Al weight ratio calculated from initial amounts of reaction precursors is 5.8, whereas Si/Al wt.% ratio by ICP-OES and XPS is 63 and 15, respectively. These differences can be explained by the presence of a certain amount of unreacted aluminum precursor which remained in the reaction mixture after the separation of the solid product.

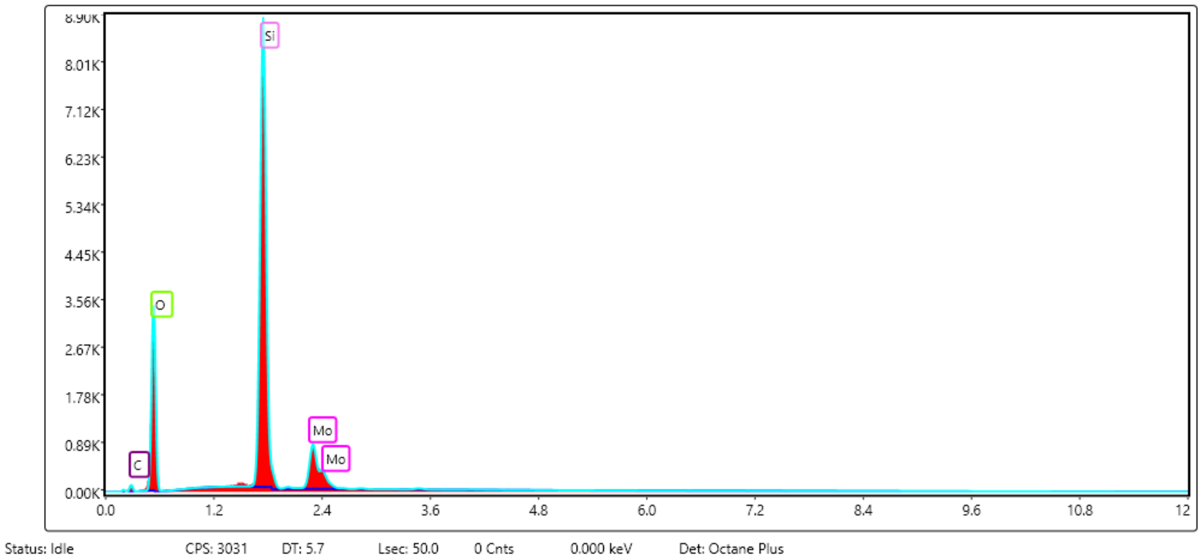

Figure S15. *SEM-EDX spectrum of 11Mo-SiO_2 sample.*

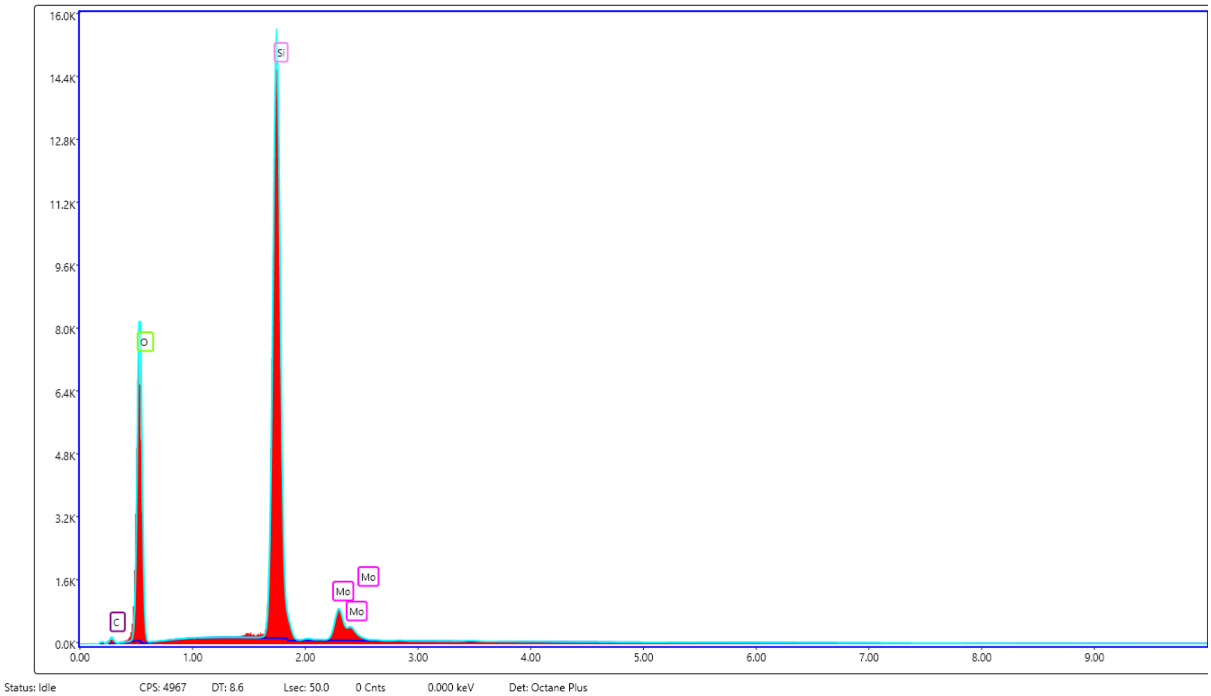

Figure S16. *SEM-EDX spectrum of 7Mo-SiO_2 sample.*

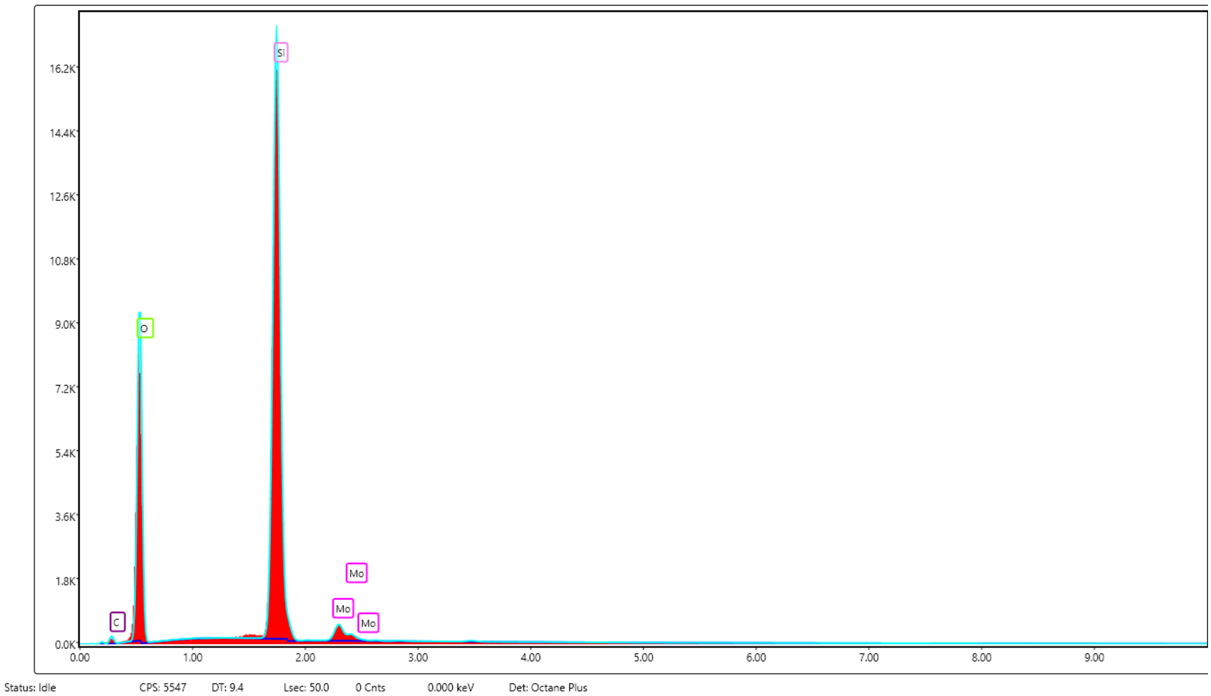

Figure S17. *SEM-EDX spectrum of the 4Mo-SiO_2 sample.*

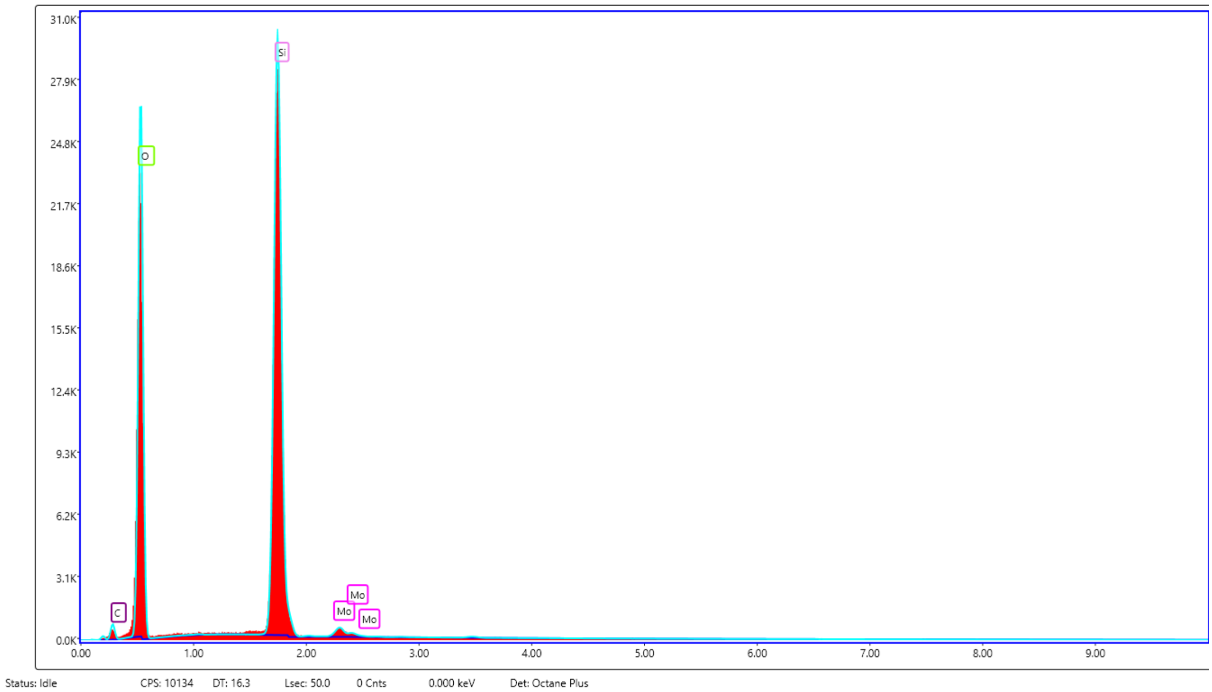

Figure S18. *SEM-EDX spectrum of the 4Mo-SiO$_2$ sample.*

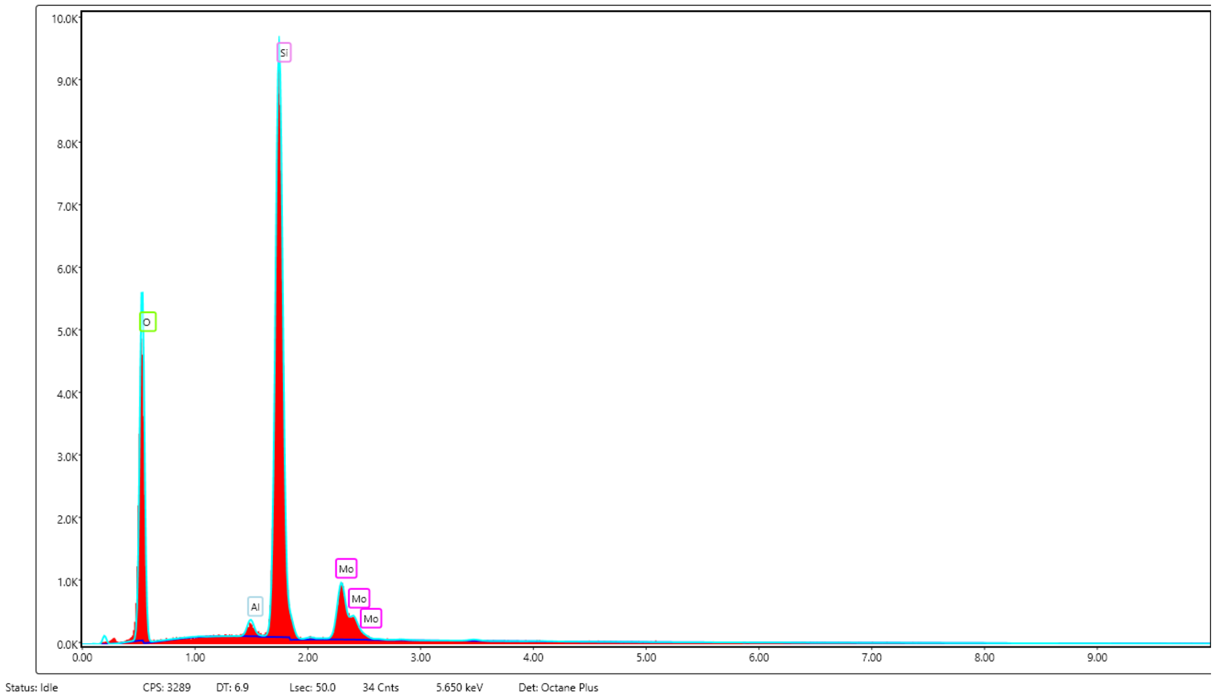

Figure S19. *SEM-EDX spectrum of Al11Mo-SiO$_2$ sample.*

S5. XPS analysis of the catalysts

Survey scans and high-resolution spectra of carbon, oxygen, silicon, and aluminum elements are given in supplementary information (Figure S20-S31).

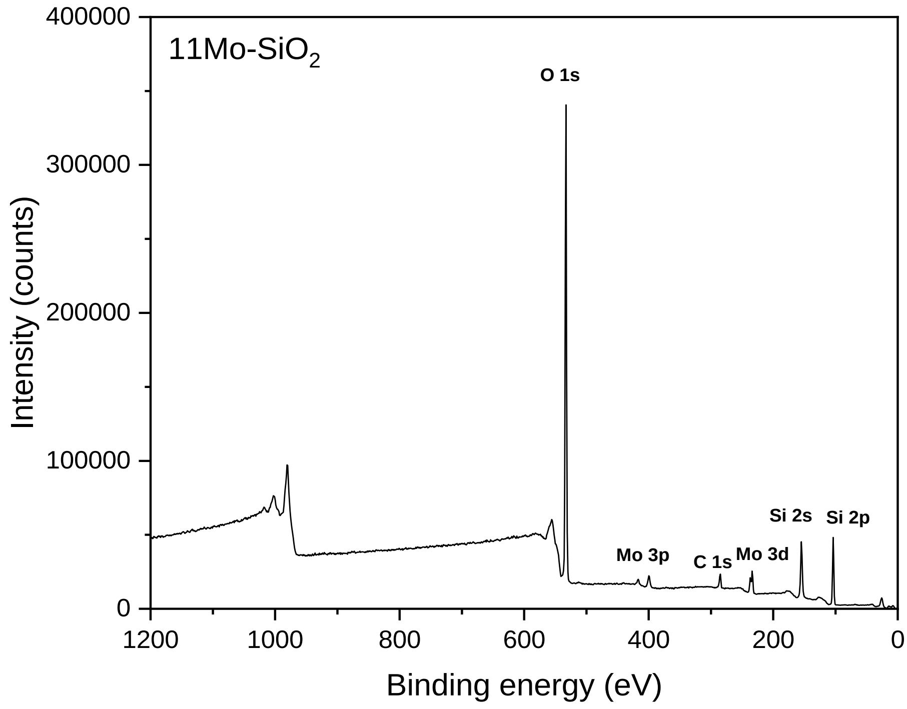

Figure S20. *Survey scan of 11Mo-SiO₂ sample.*

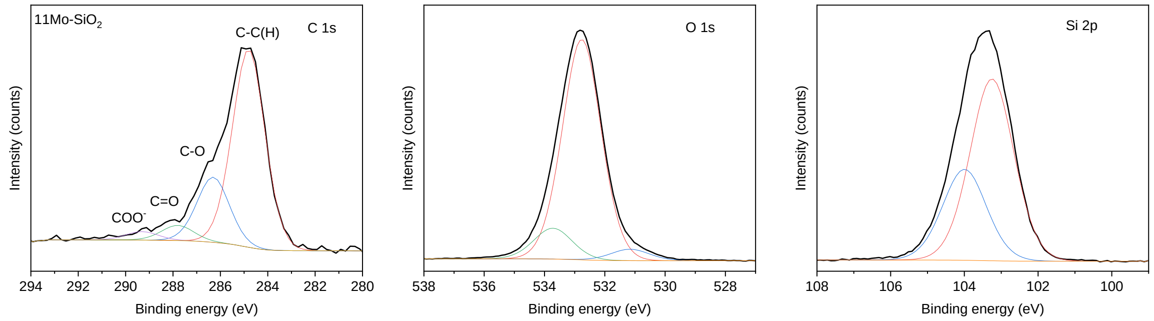

Figure S21. *11Mo-SiO$_2$: High-resolution XPS spectra of carbon, oxygen, and silicon.*

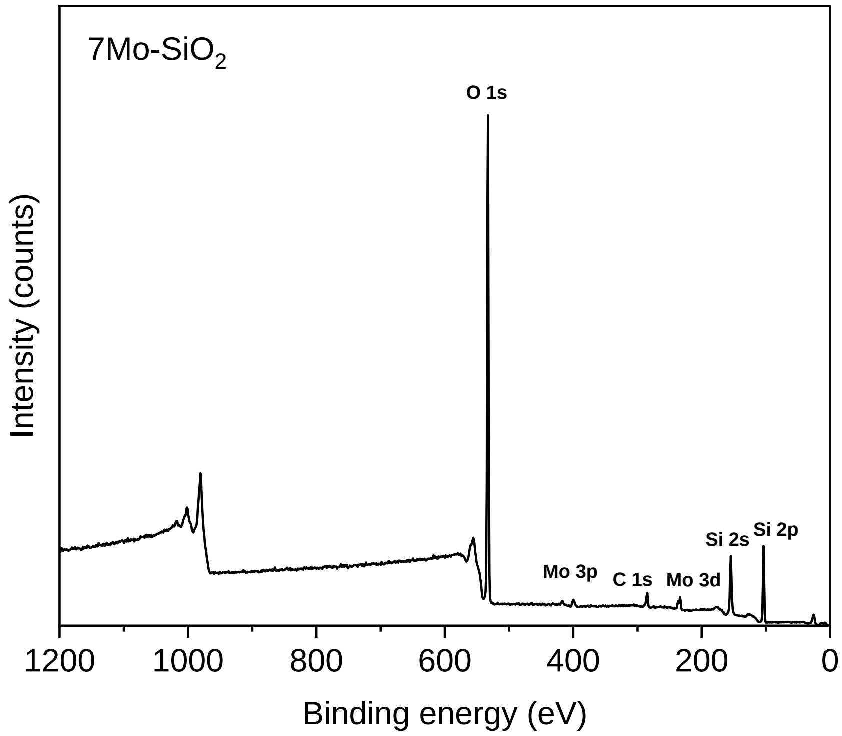

Figure S22. *Survey XPS scan of 7Mo-SiO₂ sample.*

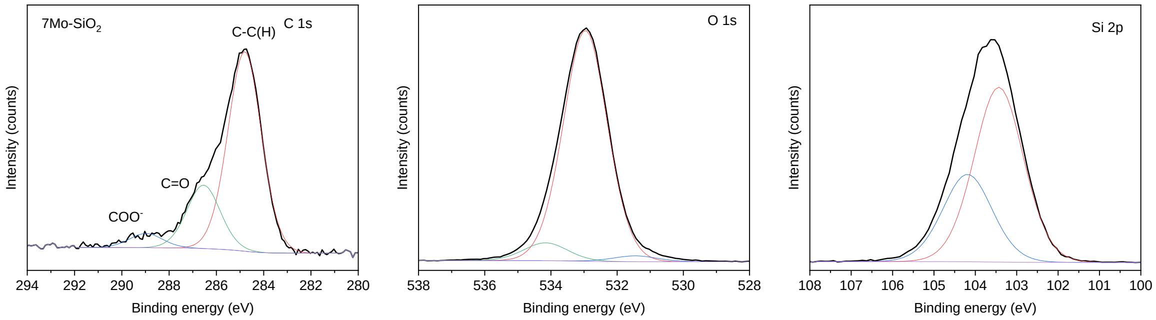

Figure S23. *7Mo-SiO_2: High-resolution XPS spectra of carbon, oxygen, and silicon.*

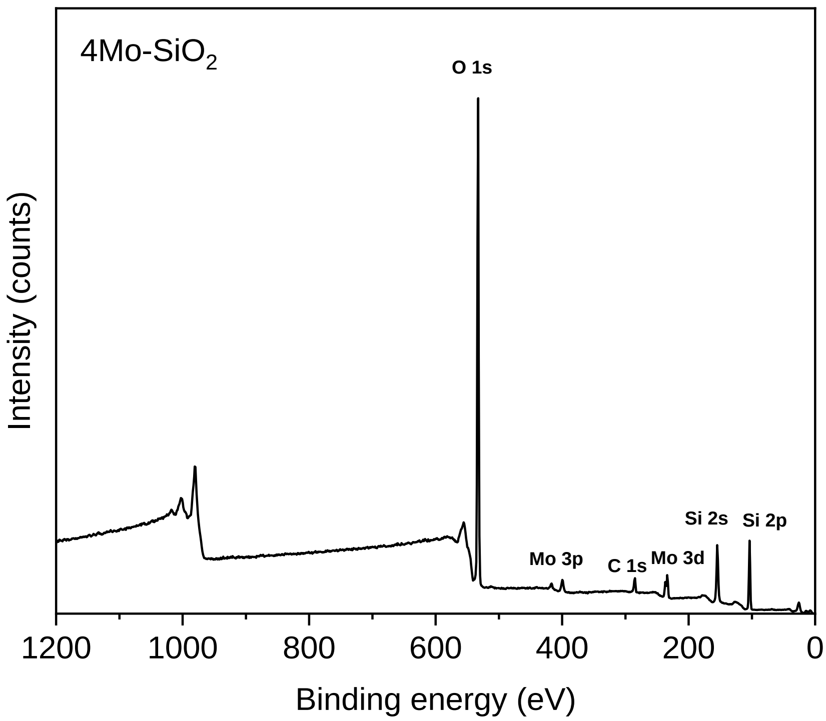

Figure S24. *Survey XPS scan of 4Mo-SiO_2 sample.*

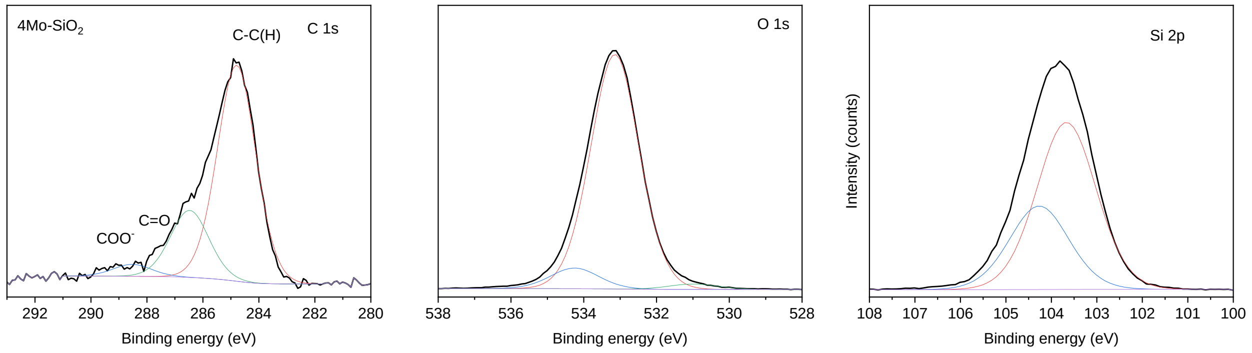

Figure S25. *4Mo-SiO_2: High-resolution XPS spectra of carbon, oxygen, and silicon.*

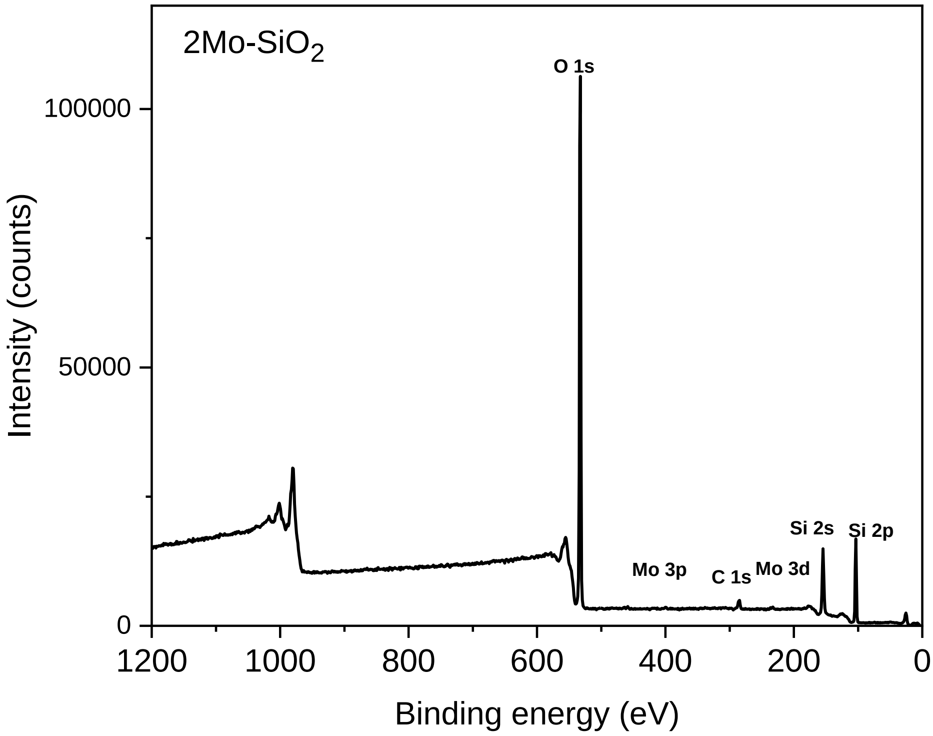

Figure S26. *Survey XPS scan of 2Mo-SiO$_2$ sample.*

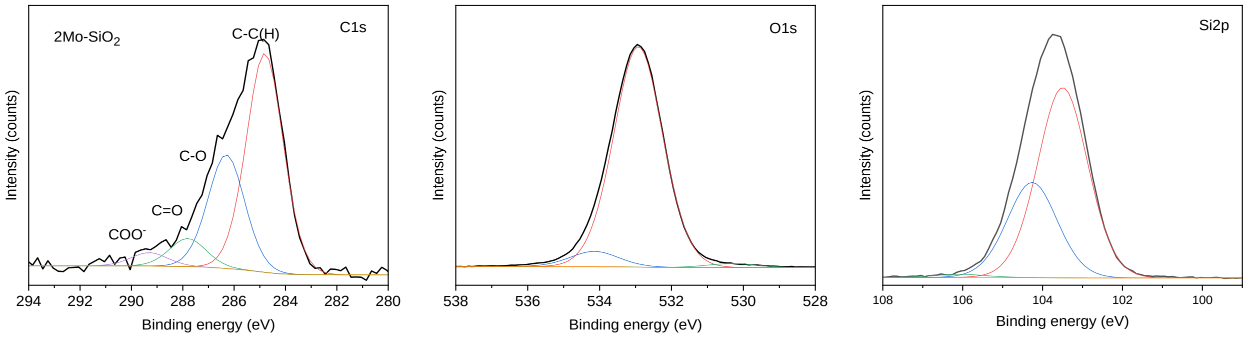

Figure S27. *2Mo-SiO$_2$: High-resolution XPS spectra of carbon, oxygen, and silicon.*

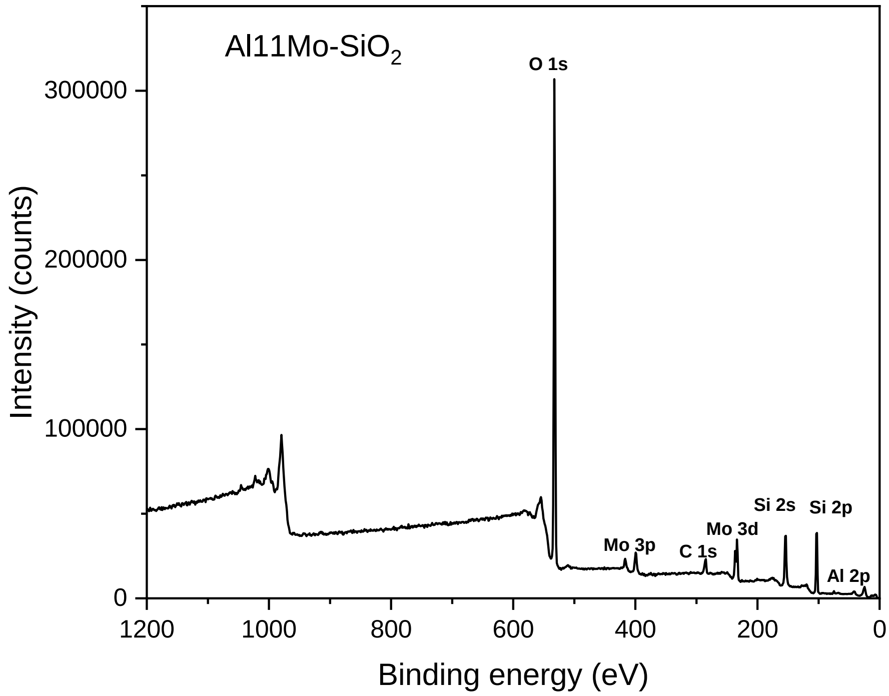

Figure S28. *Survey XPS scan of Al11Mo-SiO$_2$ sample.*

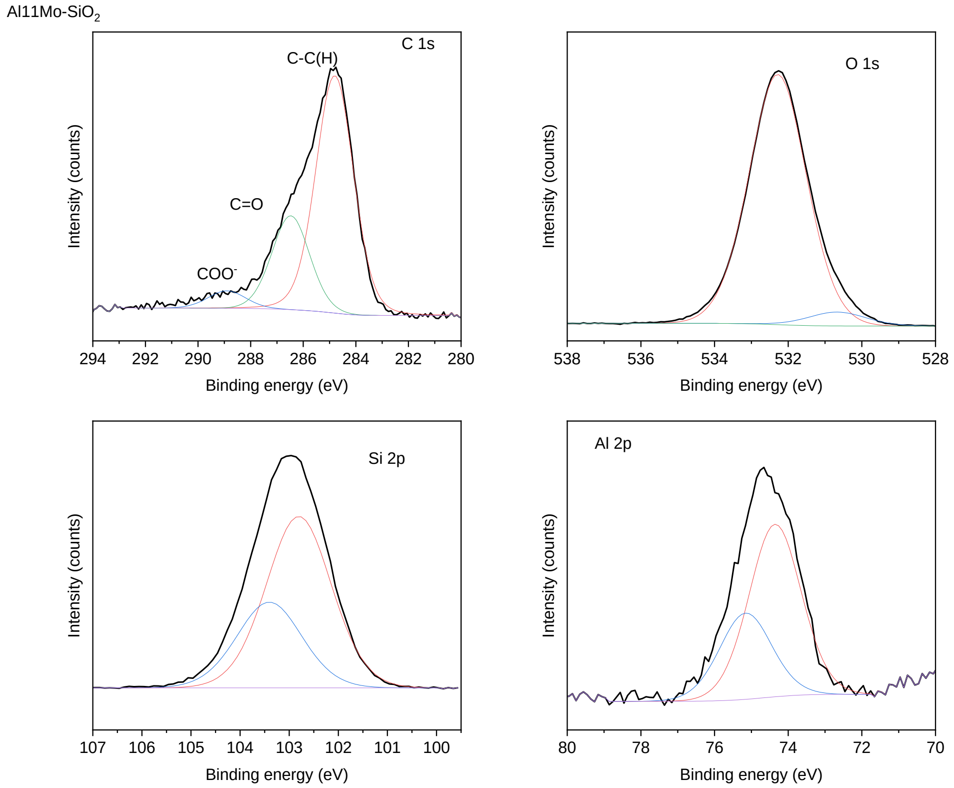

Figure S29. *Al11Mo-SiO$_2$: High-resolution XPS spectra of carbon, oxygen, aluminum, and silicon.*

The Mo 3d XPS spectra of 11Mo-SiO_2, 7Mo-SiO_2, 4Mo-SiO_2, and Al11Mo-SiO_2 samples displayed in Figures S30 and S31 show characteristic doublets of Mo $3d_{5/2}$ and Mo $3d_{3/2}$ orbitals. As demonstrated in the Mo 3d XPS spectrum of sample 11Mo-SiO_2, the signals with binding energies of 233.4 and 236.6 eV, are assigned to Mo(VI) $3d_{5/2}$ and Mo(VI) $3d_{3/2}$, respectively, indicating the presence of the Mo^{6+} state.[11,12] Furthermore, there are also contributions at BE 232.1 and 235.3 eV corresponding to Mo(V) $3d_{5/2}$ and Mo(V) $3d_{3/2}$, respectively.[13,14] Based on the Mo 3d XPS spectrum, the Mo(VI)/Mo(V) ratio for 11Mo-SiO_2 is 3.21. Similarly, the 7Mo-SiO_2 and 4Mo-SiO_2 samples with lower loading of Mo exhibited characteristic doublets of Mo $3d_{5/2}$ and Mo $3d_{3/2}$ indicating the presence of Mo(VI) and Mo(V) states.[15]

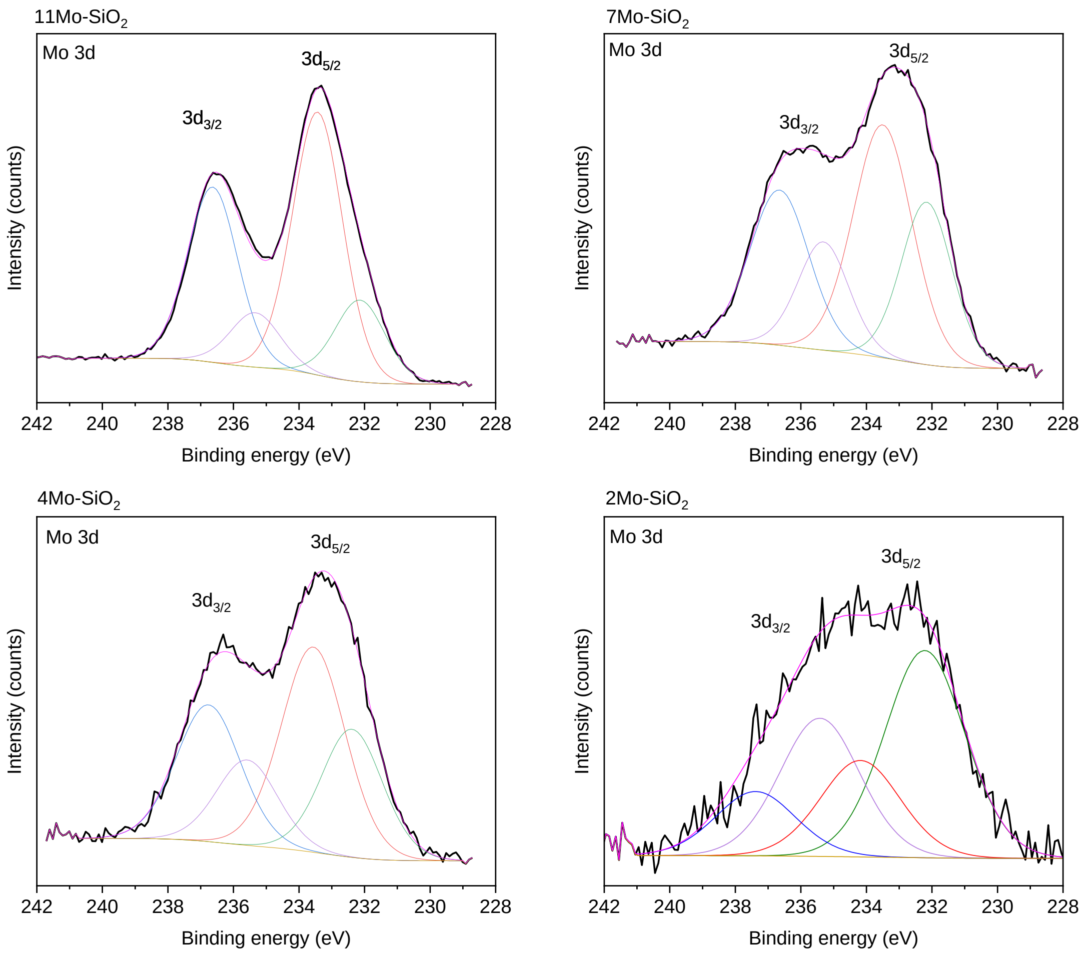

Figure S30. *High-resolution Mo 3d XPS spectra of Mo-SiO_2 microspheres.*

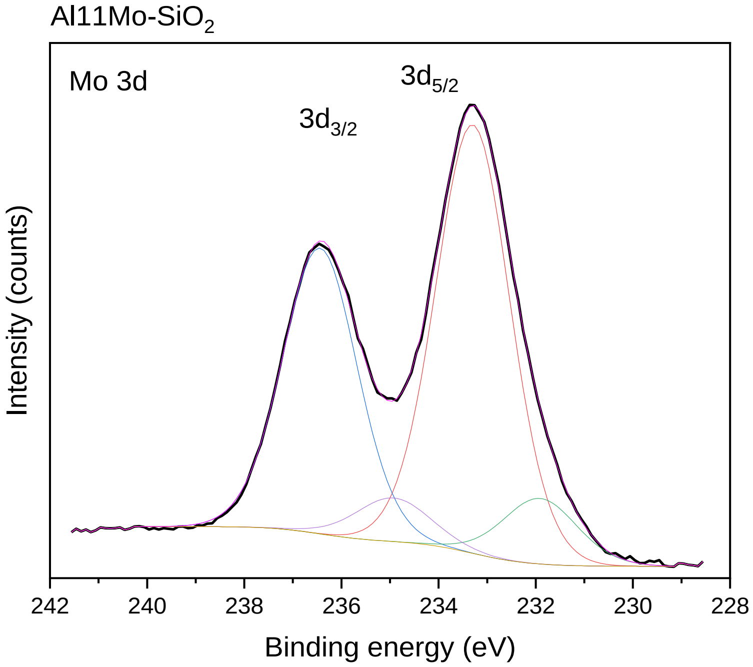

Figure S31. *High-resolution Mo 3d XPS spectrum of Al11Mo-SiO_2 microsphere sample.*

S6. Nitrogen adsorption-desorption isotherms

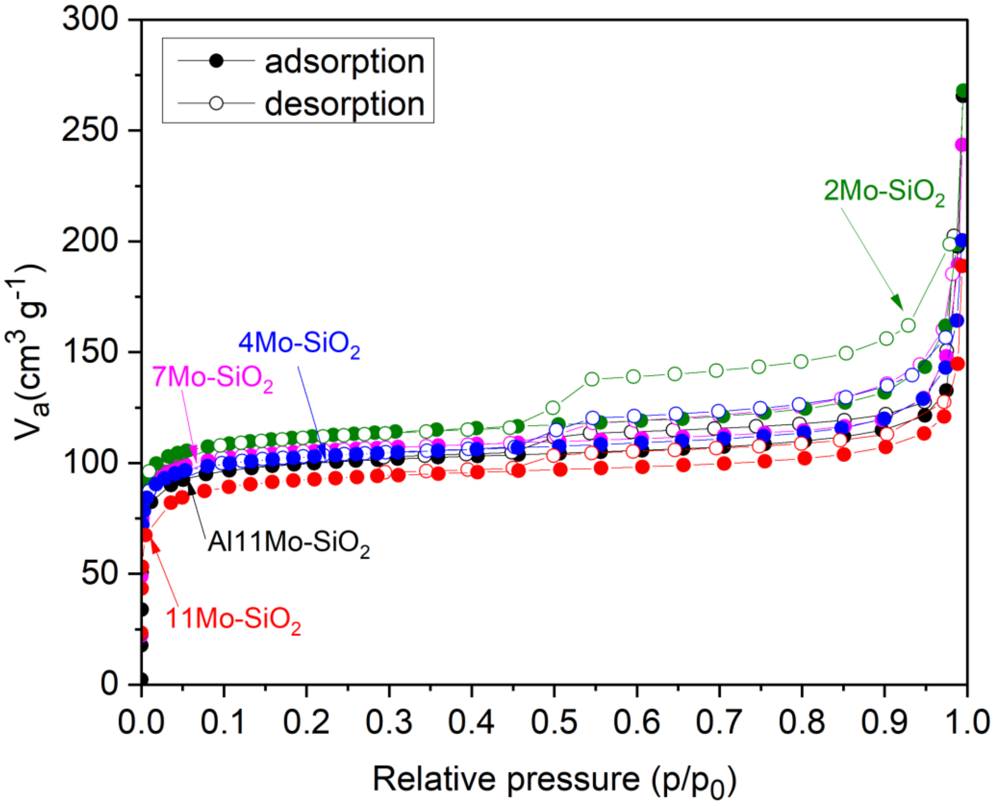

Figure S32. *Nitrogen adsorption/desorption isotherms of Mo-*SiO_2 *and Al11Mo-*SiO_2 *microspheres. They exhibit type II classification with the H4 hysteresis loop characteristic for slit-shaped pores.*[16]

Table S4. Surface areas of catalysts by the BET method. DRUV-Vis band energy values.

Samples	S_A BET [m^2 g^{-1}]	V total [cm^3 g^{-1}]	V micro [cm^3 g^{-1}]^a	Band Edge energy [eV]
11Mo-SiO_2	322	0.24	0.13	4.07
Al11Mo-SiO_2	363	0.33	0.14	4.14

^{a} *determined by t-plot method*

S7. DRUV-Vis E_g values and Raman spectroscopy

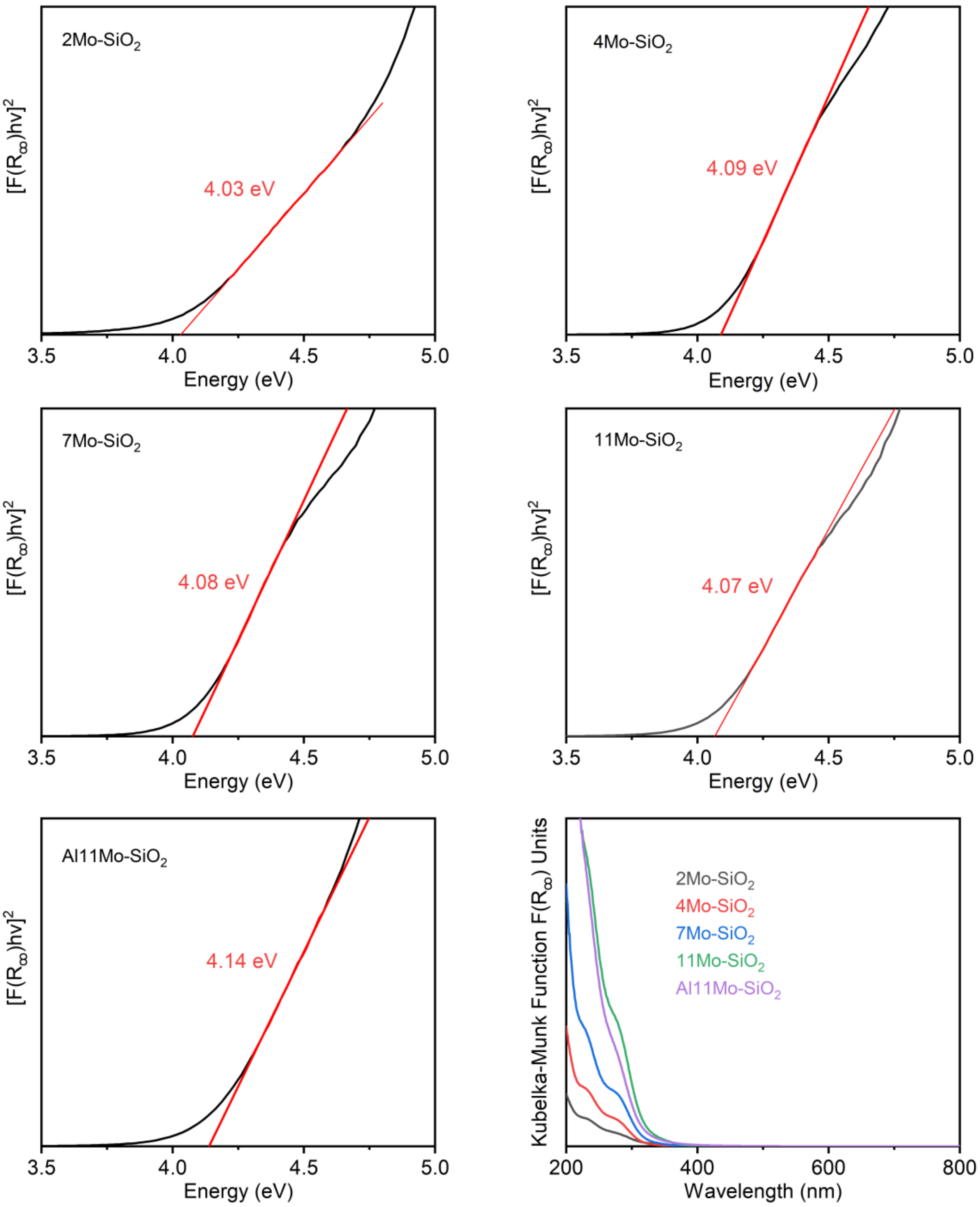

Figure S33. *In-situ DRUV-Vis band edge energy values.*

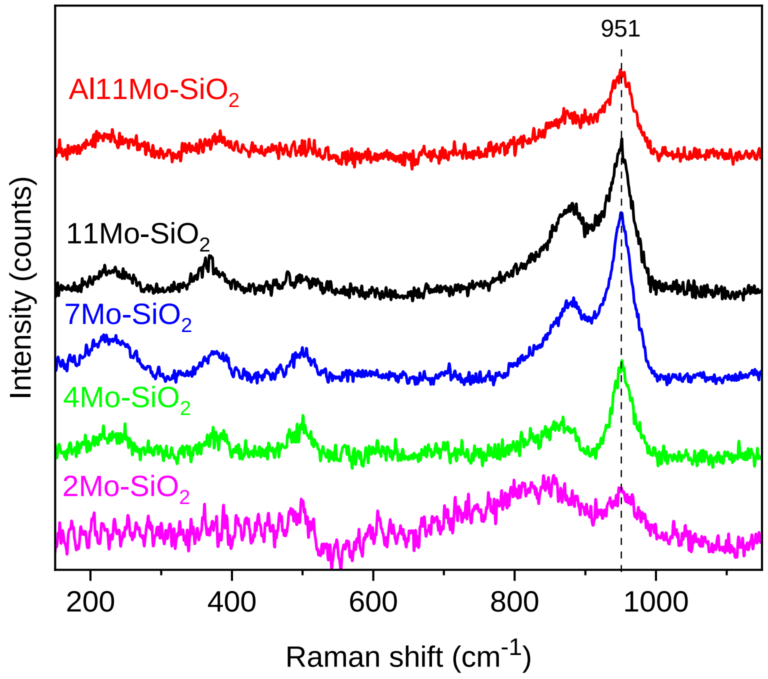

***Figure S34.** Raman spectra of Mo-SiO$_2$ and alMo-SiO$_2$ microspheres. Recorded in ambient atmosphere.*

The Raman spectra of all catalysts recorded in ambient conditions are illustrated in Figure S34. The spectra show two intense bands located at 951 and 877 cm^{-1}, which are assigned to the symmetric stretching of O=Mo=O originating from the highly dispersed tetrahedral di-oxo (Si–O)$_2$Mo(=O)$_2$ species[17,18] and vibration mode of Mo=O in oligomeric species (Mo–O–Mo), respectively.[19–23] The Raman band found at 370 cm^{-1} can be assigned to O=Mo=O bending mode.[23] It should be taken into account that these Raman bands are most likely attributed to single molybdenum-oxo species or amorphous Mo–O–Mo oligomeric species,[20,21,24] and there is no evidence of MoO$_3$ crystal phase formation in Raman spectra [25,26] confirming again the excellent dispersion of Mo oxide species. With lower Mo content, the Raman band at 951 cm^{-1} becomes more intense compared to the band at 877 cm^{-1}. This observation shows that a higher proportion of dispersed isolated tetrahedral di-oxo (Si–O)$_2$Mo(=O)$_2$ species are found with lower concentrations of Mo. For the 2Mo-SiO$_2$ sample, although the signal to noise ratio of its spectrum is lower compared to that of other samples, the intense Raman band at 951 cm^{-1} confirmed the presence of tetrahedral di-oxo (Si–O)$_2$Mo(=O)$_2$ species. Taken together, these results are in good agreement with the bands of tetrahedral di-oxo (Si–O)$_2$Mo(=O)$_2$ species in supported MoO$_x$/SiO$_2$ catalysts obtained after measurement in ambient conditions.[27]

Table S5. Characterization of MoO_x/SiO_2 catalysts.

Samples	Mo loading [a] (wt.%)	Edge Energy [b] (eV)	BET Surface Area (m^2/g)	Mo Surface Content (Mo/nm^2)
1.4% MoIWI	1.35	3.93	299	0.28
3.6% MoIWI	3.64	3.81	297	0.77
6.6% MoIWI	6.64	3.65	275	1.52
14.2% MoIWI	14.2	3.26	230	3.87

[a]. The Mo loadings of the catalysts were estimated from the ICP-OES method. [b]. Edge energy was estimated assuming an allowed transfer.[27]

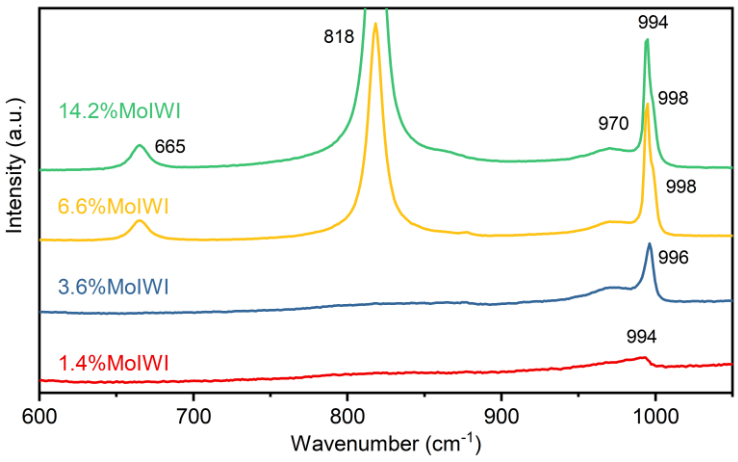

***Figure S35.** Raman spectra of dehydrated MoIWI catalysts.*

The surface MoO_x structure and speciation were studied with in-situ Raman (Figure S35), and edge energies were computed from Kubelka-Munk Function (Table S5). To focus our discussion on crystalline MoO_3 and different dispersed MoO_x species, we highlight the Raman region from 600-1050 cm^{-1}. The Raman spectra and edge energies are largely consistent with previous literature; thus, Raman peaks and edge energies are assigned to the corresponding Mo structures accordingly.[18,27–29] Starting from 1.4% MoIWI, the peak at 994 cm^{-1} is assigned to the Mo=O bond in dispersed MoO_x. With an edge energy of 3.95 eV, the 1.4% MoIWI is dominated by mono-molybdates. Increasing the Mo loading to 3.6%, we observed a weak peak at 970 cm^{-1} and a peak at 996 cm^{-1}. The new weak peak at 970 cm^{-1} may be due to the Mo-O-Si stretch of the mono-oxo[30] , the Si-OH vibration[31] , or the Mo-dioxo bond[27] . The edge energy of 3.80 eV indicates the existence of oligomerized Mo clusters on the 3.6% MoIWI catalyst. Transitioning to 6.6% and 14.2% Mo loading, the peaks at 660 cm^{-1}, 820 $cm^{-1,}$ and 995 cm^{-1} indicate the presence of MoO_3 crystalline clusters on the catalyst surface. The weak band at 970 cm^{-1} and the shoulder at 998 cm^{-1} indicate that there is still some dispersed MoO_x on the catalyst surface. The corresponding edge energies of the two samples decreased to 3.65 and 3.26 eV, respectively. These values are consistent with Raman spectra which indicate the formation of crystalline MoO_3 structure. Collectively, the Raman spectra and edge energy results show that different surface Mo structures are formed as Mo loading is increased from 1.4 to 6.6%.

S8. ToF SIMS results

Table S6. List of all Mo-based clusters identified by ToF-SIMS and considered in the formula reflecting total Mo content

Ion (cluster)	Mass (u)
MoO_3^-	145.898
MoO_4^-	161.893
$C_2H_3O_2MoO_3^-$	204.904
$SiO_2MoO_3^-$	205.855
$Mo_2O_6^-$	291.7822
$Mo_2O_7^-$	307.7866
$Mo_2O_8Al^-$	350.7492
$Mo_2O_8Si^-$	351.7515
$Mo_3O_9^-$	431.6719
$Mo_3O_{11}Al^-$	496.6374
$Mo_4O_{14}Al^-$	642.5334
$Mo_5O_{17}Al^-$	788.4374

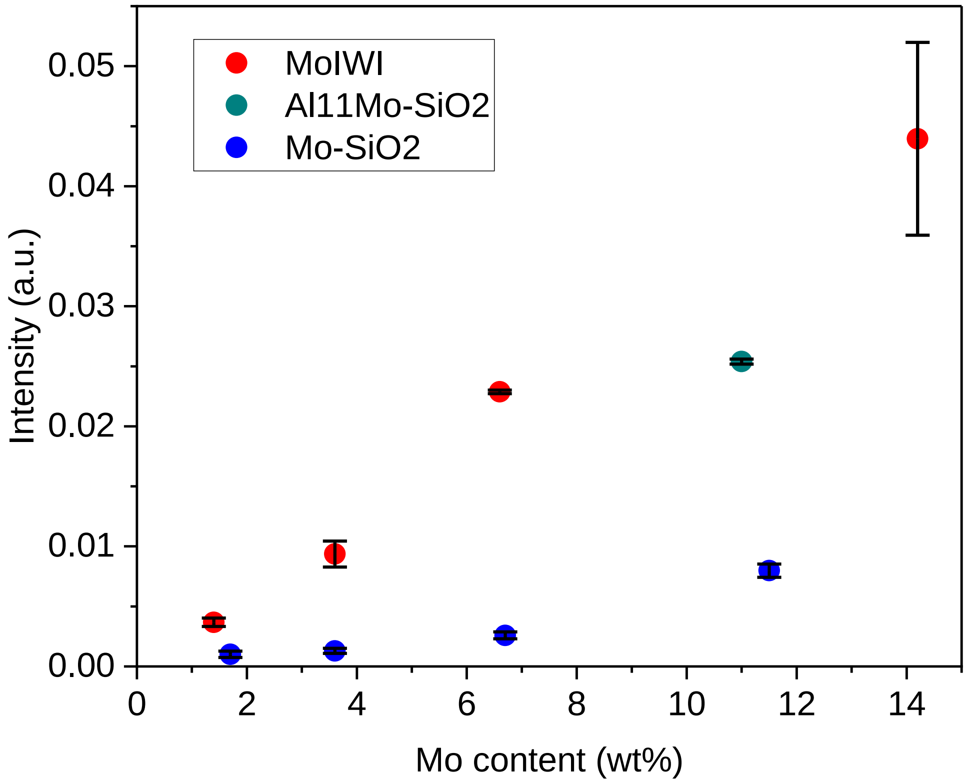

Figure S36. *The total contents of surface molybdenum species determined by ToF-SIMS technique. According to the formula: ($I_{\text{all Mo species}}/I_{\text{total count}}$), where I = intensity in counts. Mo species used for this calculation are listed in Table S5. Error bars are standard deviation of each data set (3 analyses per sample).*

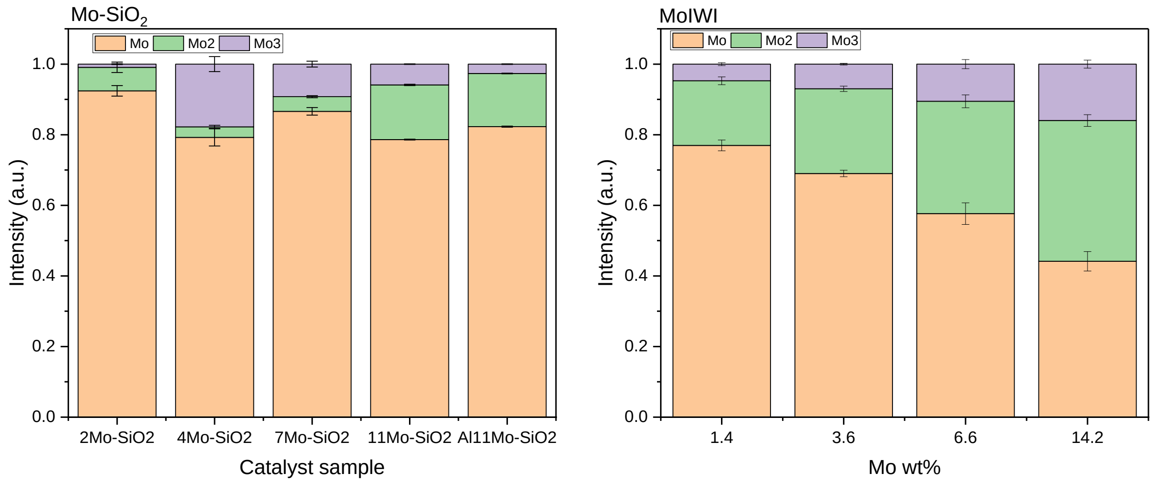

Figure S37. *The apparent contents of MoO_x, Mo_2O_x, and $Mo_3O_9^-$ species according to the formula: $I_n/(I_{MoO}+I_{Mo2O}+I_{Mo3O})$, where I = intensity of mass fragment in counts and n = MoO_x, Mo_2O_x, or $Mo_3O_9^-$.*

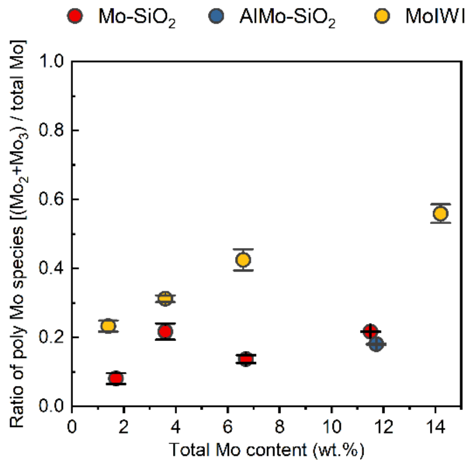

Figure S38. *The graph reflects the (Mo_2+ Mo_3)/ total Mo species ratio on the surface of prepared catalysts. (Mo_2+ Mo_3) = sum of ions: $Mo_2O_6^-$, $Mo_2O_7^-$, $Mo_2O_8Al^-$, $Mo_2O_8Si^-$, $Mo_3O_9^-$, $Mo_3O_{11}Al^-$, $Mo_4O_{14}Al^-$, $Mo_5O_{17}Al^-$ (Table S5).*

S9. In-situ FTIR for pyridine adsorption on Al11Mo-SiO_2

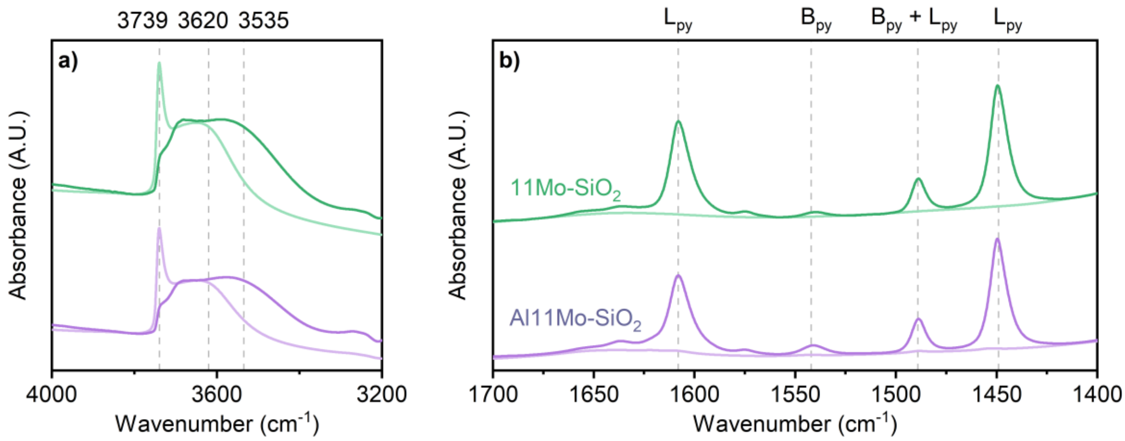

Figure S39. *In-situ FTIR spectra of 11Mo-SiO_2 and Al11Mo-SiO_2 microspheres before (light color) and after (dark color) pyridine adsorption, a) in the region 3200-4000 cm^{-1}, and b)* in the region 1400-1700 cm^{-1}.

S10. Supplementary of the reactivity results

Table S7. Steady state rate, contact time, and steady state propylene conversion for each catalyst bed.

Samples:	Rate ($\mu mol/g_{cat}s$)	m_{cat} (mg)	Contact time (min)	Propylene Conversion (%)
2Mo-SiO_2	10.0	13.3	0.26	0.60
	9.91	13.7	0.26	0.60
4Mo-SiO_2	43.2	20.8	0.40	4.04
	38.9	22.0	0.42	3.76
	41.3	36.0	0.70	6.67
	39.3	10.6	0.20	1.87
7Mo-SiO_2	42.6	21.8	0.42	4.16
	43.0	20.1	0.39	3.91
11Mo-SiO_2	21.7	20.1	0.39	1.98
	22.0	31.8	0.61	3.09
	22.1	26.8	0.52	2.59
	21.9	21.0	0.41	2.09
Al11Mo-SiO_2	13.7	34.2	0.66	2.02
2MoIWI	0.10	60.3	1.16	0.03
4MoIWI	0.88	56.2	1.09	0.21
7MoIWI	3.51	32.5	0.63	0.50
14MoIWI	1.74	33.2	0.64	0.25

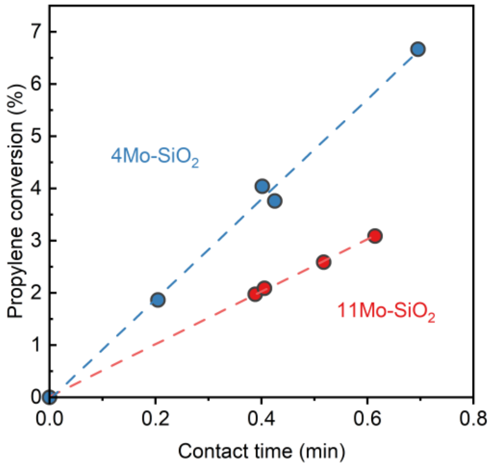

Figure S40. *Propylene conversion over 4Mo-SiO_2 and 11Mo-SiO_2 as a function of contact time. Conditions: 50% propylene in a helium balance, 50 mL/min total flowrate, at 200 °C.*

Steady state rates of all samples were obtained at ≤ 7% propylene conversion. In this range (Figure S40), propylene conversion increases linearly with contact time (τ)—defined here as the inverse of weight hourly-space-velocity (Eq. (S1))—indicating that the differential condition approximation is reasonable.

$$\tau(\text{min}) = \frac{\text{mass}_{cat}\,(\text{g})}{\text{mass flowrate of propylene}\,(\text{g}/\text{min})} \quad \text{(Eq. S1)}$$

Direct tests for transport limitations (the Madon-Boudart test) present challenges in assessing structure-sensitive reactions, as alterations in molybdenum loading may concurrently impact the structure and reactivity of supported molybdates (the pre-active sites).

To address this concern and ensure clear determination of the reaction rate, we estimated theoretical criteria for maintaining kinetic control. The threshold criteria were evaluated based on the maximum propylene metathesis rate (5×10^{-5} mol/g_{cat}·s). Refer to Table S8 for a summary of the value used to estimate mass and heat transfer criteria.

Table S8. Summary of the value for computing mass and heat transfer criteria.

Physical quantity	Symbol	Value (2 s.f.)	Notes
Catalyst particle radius	r_p	1.0×10^{-7} m	Figure S13
Average pore diameter for catalyst	d_p	2.0×10^{-9} m	
Bulk propylene concentration	C_A	$10\ \mathrm{mol/m^3}$	From ideal gas law
Tubing inner radius	r_t	2.3×10^{-3} m	
Total gas flow rate	Q	$1.4 \times 10^{-6}\ \mathrm{m^3/s}$	Converted from STP to reaction T
Density of gas mixture	ρ	$0.59\ \mathrm{kg/m^3}$	From ideal gas law assuming equimolar He and propylene mixture
Viscosity of gas mixture	μ	$2.0 \times 10^{-5}\ \mathrm{Pa \cdot s}$	Estimated from arithmetic mean of pure helium and propylene viscosities
Reynolds number	Re	11	
Schmidt number	Sc	0.34	
Bulk gas diffusivity	D_b	$1.0 \times 10^{-4}\ \mathrm{m^2/s}$	Typical value for ideal gas mixtures
Mass transfer coefficient	k_m	$2.2 \times 10^{3}\ \mathrm{m/s}$	$Sh = k_m (2r_p)/D_{AB} = 2 + Re^{1/2} \cdot Sc^{1/3}$ for laminar flow over a sphere
Mean velocity	ῡ	490 m/s	$[(8k_BT)/(\pi m)]^{1/2}$
Knudsen diffusivity	D_K	$3.26 \times 10^{-7}\ \mathrm{m^2/s}$	$\bar{\upsilon} d_p/3$
Effective diffusivity	D_e	$3.25 \times 10^{-7}\ \mathrm{m^2/s}$	$D_e = 1/(1/D_b + 1/D_K)$
Thermal conductivity of gas mixture	k_g	$0.10\ \mathrm{W/m \cdot K}$	
Heat capacity of gas mixture	C_p	$3.7 \times 10^{3}\ \mathrm{J/kg \cdot K}$	Weighted average of pure helium and propylene heat capacities
Prandtl number	Pr	0.76	
Heat transfer coefficient	h	$2.5 \times 10^{6}\ \mathrm{W/m^2 \cdot K}$	$Nu = h(2r_p)/k_g = 2 + Re^{1/2} \cdot Pr^{1/3}$ for laminar flow over a sphere
Density of catalyst support	ρ_s	$1.6 \times 10^{3}\ \mathrm{kg/m^3}$	Approximated as density of crystalline silica multiplied by porosity of 0.4
Observed reaction rate (volumetric)	r’’’	$80\ \mathrm{mol/m^3_{cat} \cdot s}$	From $r = 5\times10^{-5}$ mol/g_{cat}·s
Thermal conductivity of support	k	$1\ \mathrm{W/m \cdot K}$	
Reaction enthalpy	$\lvert \Delta H_r \rvert$	$6\ \mathrm{kJ/mol}$	
Apparent activation energy	E_a	$60\ \mathrm{kJ/mol}$	

The threshold criteria were computed via Eq. S2-S5. Eq. S2 was taken from *Kinetics of Catalytic Reactions* by Vannice,[32] and equation S3-S5 were taken from *The Microkinetics of Heterogeneous Catalysis* by Dumesic[33]. All the estimated criteria are significantly below the limitation criteria, indicating the reported rates (< 5×10^{-5} mol/$g_{cat}\cdot$s) are free of mass and heat transfer limitations.

Weisz-Prater Criteria for excluding intraparticle mass transfer limitations:

$$\frac{r''' \times r_P^2}{C_A \times D_e} = 2.5\times10^{-7} < 0.3 \qquad \text{(Eq. S2)}$$

Criteria for excluding interphase mass transfer limitations:

$$\frac{r''' r_P}{C_A k_m} = 3.7\times10^{-10} < 0.15 \qquad \text{(Eq. S3)}$$

Criteria for excluding intraparticle Heat Transfer Limitations

$$\frac{|\Delta H_r| r''' r_P^2}{k_g T} \times \frac{E_A}{RT} = 1.6\times10^{-9} < 0.75 \qquad \text{(Eq. S4)}$$

Criteria for excluding interphase heat transfer limitations:

$$\frac{|\Delta H_r| \times r''' \times r_P}{hT} \times \frac{E_A}{RT} = 6.2\times10^{-10} < 0.15 \qquad \text{(Eq. S5)}$$

S11. Propylene metathesis rate of Mo-SiO_2 microspheres as a function of time on stream

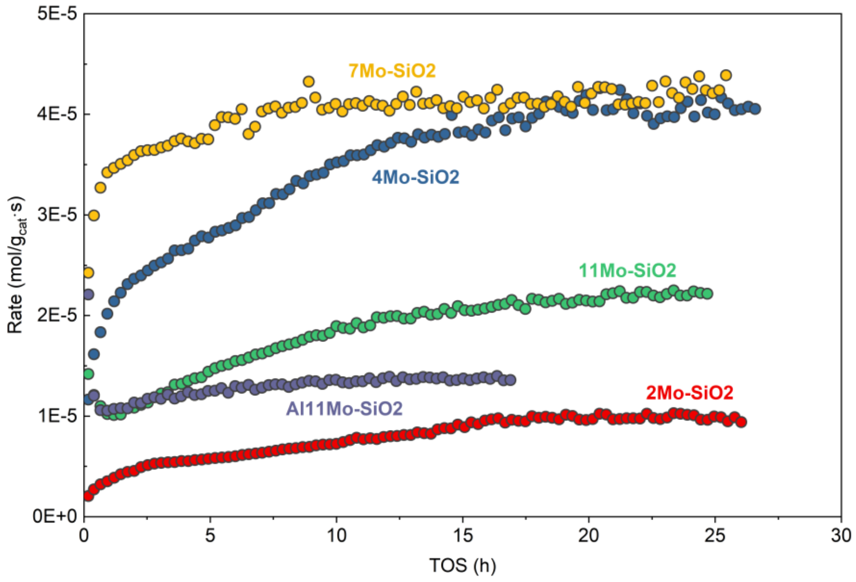

Figure S41. *Catalyst mass normalized propylene metathesis rate of Mo-SiO_2 and Al11Mo-SiO_2 microspheres as a function of time on stream (TOS). Conditions: 20 mg catalyst, 50% propylene in a helium balance, 50 mL/min total flowrate, at 200 °C.*

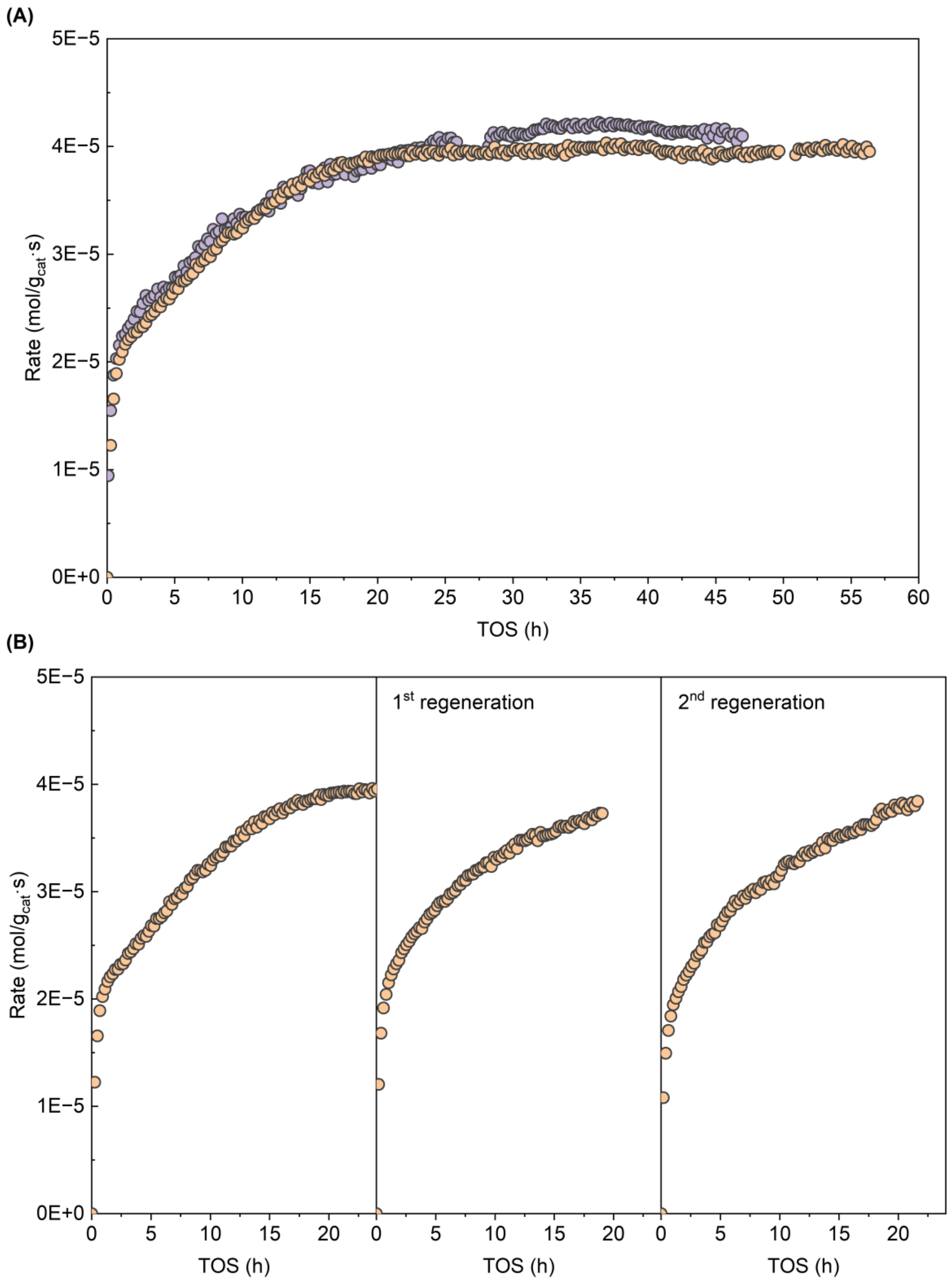

Figure S42. (A) *Stability test of two catalyst beds and* ***(B)*** *regeneration test of one catalyst bed with 4Mo-SiO$_2$. Regeneration was performed using the same pretreatment steps for fresh catalyst. Conditions: 10-30 mg catalyst, 50% propylene in a helium balance, 50 mL/min total flowrate, at 200 °C.*

S12. The site renewal and decay cycle

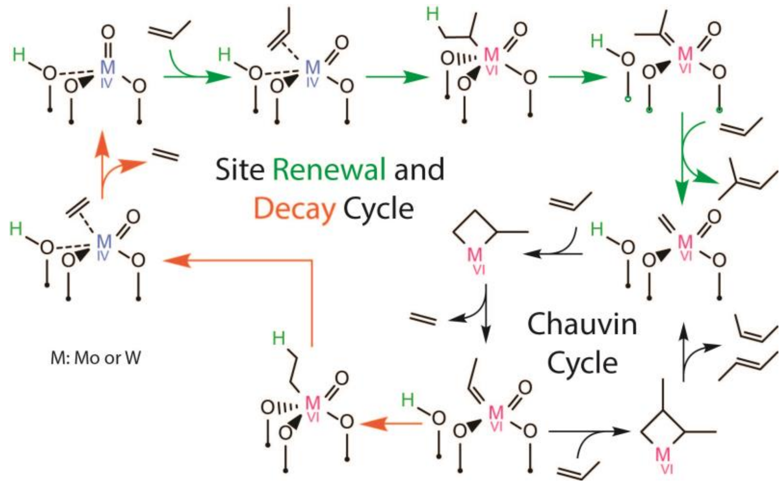

Scheme S1. *The 1,2-proton shift mechanism from partially reduced Mo(IV) oxo to Mo(VI) oxo propylidene.*

In a recent study, we demonstrated that after reaching the steady state for propylene self-metathesis, the surface W and Mo alkylidene structures on the catalyst surface are not stable when the surface is purged with pure helium.[34] To explain this dynamic change in metal alkylidenes, we proposed a site renewal and decay cycle based on the well-documented 1,2-proton shift mechanism (Scheme S1). [35–38] Furthermore, we utilized this site renewal and decay cycle to drastically boost the steady state metathesis rate by co-feeding a highly substituted olefin, namely 2,3-dimethyl-1-butene (i-4ME).[34] We showed that the co-fed i-4ME can serve as a proton shuttle, facilitating proton transfer during the site renewal steps. We proposed that the promotional effect of co-feeding i-4ME is mainly attributed to the increased quantity of metal alkylidenes (the authentic active site for olefin metathesis) on the catalyst surface resulting from the promoted site renewal steps. These findings underscore the crucial role of surface protons in the formation of metal alkylidene structures for propylene self-metathesis.

S13. Influence of product co-feeding on the steady state rates.

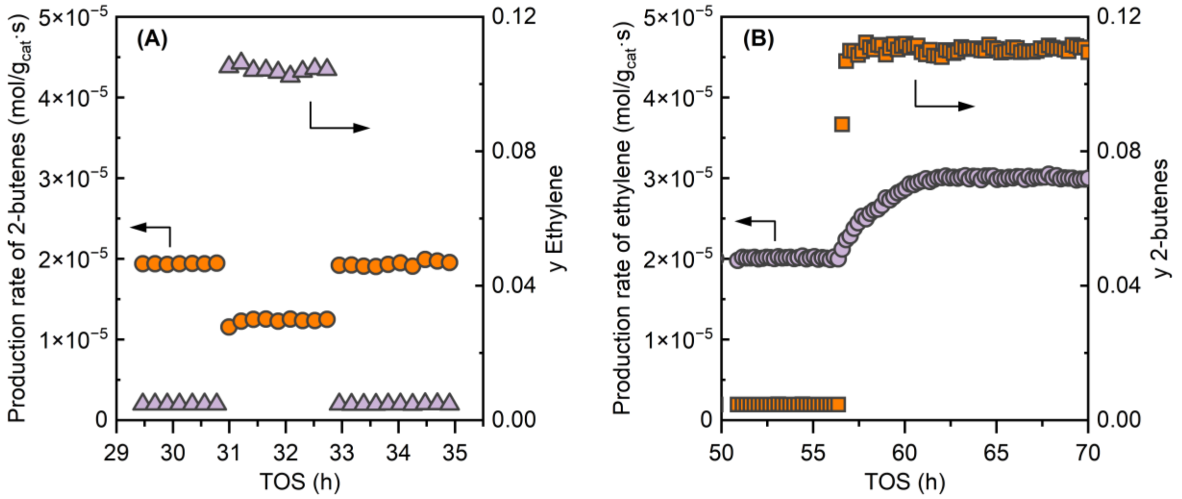

Figure S43. *Influence of* ***(A)*** *10% ethylene and* ***(B)*** *10% 2-butenes co-feeding on the steady state rates of propylene metathesis over 4Mo-SiO$_2$. Conditions: 10mg 4Mo-SiO$_2$, 50% propylene in a helium balance, 50 mL/min total flowrate, at 200 °C.*

Co-feeding 10% ethylene to the steady state propylene self-metathesis over 4Mo-SiO$_2$, we observed a 36% decrease in the production rate of 2-butenes. However, co-feeding 10% 2-butenes generated a promotional effect (1.5x) in the production of ethylene. This promotional effect is similar to the promotional effect observed on supported MoO$_x$/SiO$_2$,[34] under is currently under investigation.

S14. References

(1) Skoda, D.; Hanulikova, B.; Styskalik, A.; Vykoukal, V.; Machac, P.; Urbanek, P.; Domincova Bergerova, E.; Simonikova, L.; Kuritka, I. Non-Aqueous Synthesis of Homogeneous Molybdenum Silicate Microspheres and Their Application as Heterogeneous Catalysts in Olefin Epoxidation and Selective Aniline Oxidation. *J. Ind. Eng. Chem.* **2022**, *107*, 320–332. https://doi.org/10.1016/j.jiec.2021.12.001.

(2) Dhumal, N. R.; Singh, M. P.; Anderson, J. A.; Kiefer, J.; Kim, H. J. Molecular Interactions of a Cu-Based Metal–Organic Framework with a Confined Imidazolium-Based Ionic Liquid: A Combined Density Functional Theory and Experimental Vibrational Spectroscopy Study. *J. Phys. Chem. C* **2016**, *120* (6), 3295–3304. https://doi.org/10.1021/acs.jpcc.5b10123.

(3) Huang, Y.; Jiang, Z.; Schwieger, W. Vibrational Spectroscopic Studies of Layered Silicates. *Chem. Mater.* **1999**, *11* (5), 1210–1217. https://doi.org/10.1021/cm980403m.

(4) Adam, F.; Iqbal, A. Silica Supported Amorphous Molybdenum Catalysts Prepared via Sol-Gel Method and Its Catalytic Activity. *Microporous Mesoporous Mater.* **2011**, *141* (1–3), 119–127. https://doi.org/10.1016/j.micromeso.2010.10.037.

(5) Sarkheil, M.; Lashanizadegan, M. New Magnetic Supported Hydrazone Schiff Base Dioxomolybdenum (VI) Complex: An Efficient Nanocatalyst for Epoxidation of Cyclooctene and Norbornene. *Appl. Organomet. Chem.* **2018**, *32* (9), e4459. https://doi.org/10.1002/aoc.4459.

(6) Shen, Y.; Jiang, P.; Zhang, J.; Bian, G.; Zhang, P.; Dong, Y.; Zhang, W. Highly Dispersed Molybdenum Incorporated Hollow Mesoporous Silica Spheres as an Efficient Catalyst on Epoxidation of Olefins. *Mol. Catal.* **2017**, *433*, 212–223. https://doi.org/10.1016/j.mcat.2016.12.011.

(7) Ono, T.; Anpo, M.; Kubokawa, Y. Catalytic Activity and Structure of MoO3 Highly Dispersed on SiO2. *J. Phys. Chem.* **1986**, *90* (20), 4780–4784. https://doi.org/10.1021/j100411a014.

(8) Protsak, I. S.; Morozov, Y. M.; Dong, W.; Le, Z.; Zhang, D.; Henderson, I. M. A 29Si, 1H, and 13C Solid-State NMR Study on the Surface Species of Various Depolymerized Organosiloxanes at Silica Surface. *Nanoscale Res. Lett.* **2019**, *14* (1), 160. https://doi.org/10.1186/s11671-019-2982-2.

(9) Dubray, F.; Dib, E.; Medeiros-Costa, I.; Aquino, C.; Minoux, D.; van Daele, S.; Nesterenko, N.; Gilson, J.-P.; Mintova, S. The Challenge of Silanol Species Characterization in Zeolites. *Inorg. Chem. Front.* **2022**, *9* (6), 1125–1133. https://doi.org/10.1039/D1QI01483H.

(10) Valla, M.; Rossini, A. J.; Caillot, M.; Chizallet, C.; Raybaud, P.; Digne, M.; Chaumonnot, A.; Lesage, A.; Emsley, L.; van Bokhoven, J. A.; Copéret, C. Atomic Description of the Interface between Silica and Alumina in Aluminosilicates through Dynamic Nuclear Polarization Surface-Enhanced NMR Spectroscopy and First-Principles Calculations. *J. Am. Chem. Soc.* **2015**, *137* (33), 10710–10719. https://doi.org/10.1021/jacs.5b06134.

(11) Xie, S.; Cao, D.; She, Y.; Wang, H.; Shi, J.-W.; Leung, M. K. H.; Niu, C. Atomic Layer Deposition of TiO 2 Shells on MoO 3 Nanobelts Allowing Enhanced Lithium Storage Performance. *Chem. Commun.* **2018**, *54* (56), 7782–7785. https://doi.org/10.1039/C8CC04282A.

(12) Ramanathan, A.; Wu, J. F.; Maheswari, R.; Hu, Y.; Subramaniam, B. Synthesis of Molybdenum-Incorporated Mesoporous Silicates by Evaporation-Induced Self-Assembly: Insights into Surface Oxide Species and Corresponding Olefin Metathesis Activity. *Microporous Mesoporous Mater.* **2017**, *245*, 118–125. https://doi.org/10.1016/j.micromeso.2017.03.001.

(13) Dong, W. J.; Ham, J.; Jung, G. H.; Son, J. H.; Lee, J.-L. Ultrafast Laser-Assisted Synthesis of

Hydrogenated Molybdenum Oxides for Flexible Organic Solar Cells. *J. Mater. Chem. A* **2016**, *4* (13), 4755–4762. https://doi.org/10.1039/C5TA10032A.

(14) Rellán-Piñeiro, M.; López, N. One Oxygen Vacancy, Two Charge States: Characterization of Reduced α-MoO 3 (010) through Theoretical Methods. *J. Phys. Chem. Lett.* **2018**, *9* (10), 2568–2573. https://doi.org/10.1021/acs.jpclett.8b00536.

(15) Lin, C.; Tao, K.; Yu, H.; Hua, D.; Zhou, S. Enhanced Catalytic Performance of Molybdenum-Doped Mesoporous SBA-15 for Metathesis of 1-Butene and Ethene to Propene. *Catal. Sci. Technol.* **2014**, *4* (11), 4010–4019. https://doi.org/10.1039/C4CY00652F.

(16) Thommes, M. Physical Adsorption Characterization of Nanoporous Materials. *Chemie Ing. Tech.* **2010**, *82* (7), 1059–1073. https://doi.org/10.1002/cite.201000064.

(17) Handzlik, J.; Sautet, P. Structure of Isolated Molybdenum(VI) Oxide Species on γ-Alumina: A Periodic Density Functional Theory Study. *J. Phys. Chem. C* **2008**, *112* (37), 14456–14463. https://doi.org/10.1021/jp802372e.

(18) Lee, E. L.; Wachs, I. E. In Situ Spectroscopic Investigation of the Molecular and Electronic Structures of SiO 2 Supported Surface Metal Oxides. *J. Phys. Chem. C* **2007**, *111* (39), 14410–14425. https://doi.org/10.1021/jp0735482.

(19) Uchagawkar, A.; Ramanathan, A.; Hu, Y.; Subramaniam, B. Highly Dispersed Molybdenum Containing Mesoporous Silicate (Mo-TUD-1) for Olefin Metathesis. *Catal. Today* **2020**, *343* (March 2019), 215–225. https://doi.org/10.1016/j.cattod.2019.03.073.

(20) Gong, L.; Haur, S. C. On Demand Rapid Patterning of Colored Amorphous Molybdenum Oxide Using a Focused Laser Beam. *J. Mater. Chem. C* **2017**, *5* (8), 2090–2097. https://doi.org/10.1039/C6TC04580D.

(21) Ajito, K.; Nagahara, L. A.; Tryk, D. A.; Hashimoto, K.; Fujishima, A. Study of the Photochromic Properties of Amorphous MoO3 Films Using Raman Microscopy. *J. Phys. Chem.* **1995**, *99* (44), 16383–16388. https://doi.org/10.1021/j100044a028.

(22) Ciocan, C. E.; Dumitriu, E.; Cacciaguerra, T.; Fajula, F.; Hulea, V. New Approach for Synthesis of Mo-Containing LDH Based Catalysts. *Catal. Today* **2012**, *198* (1), 239–245. https://doi.org/10.1016/j.cattod.2012.04.071.

(23) Liu, Y.; Li, J.; Das, A.; Kim, H.; Jones, L. O.; Ma, Q.; Bedzyk, M. J.; Schatz, G. C.; Kratish, Y.; Marks, T. J. Synthesis and Structure–Activity Characterization of a Single-Site MoO 2 Catalytic Center Anchored on Reduced Graphene Oxide. *J. Am. Chem. Soc.* **2021**, *143* (51), 21532–21540. https://doi.org/10.1021/jacs.1c07236.

(24) Chakrabarti, A.; Wachs, I. E. Molecular Structure–Reactivity Relationships for Olefin Metathesis by Al 2 O 3 -Supported Surface MoO x Sites. *ACS Catal.* **2018**, *8* (2), 949–959. https://doi.org/10.1021/acscatal.7b03598.

(25) Dieterle, M.; Weinberg, G.; Mestl, G. Raman Spectroscopy of Molybdenum Oxides. *Phys. Chem. Chem. Phys.* **2002**, *4* (5), 812–821. https://doi.org/10.1039/b107012f.

(26) Zhang, B.; Ford, M. E.; Ream, E.; Wachs, I. E. Olefin Metathesis over Supported MoO x Catalysts: Influence of the Oxide Support. *Catal. Sci. Technol.* **2023**, *13* (1), 217–225. https://doi.org/10.1039/D2CY01612E.

(27) Tian, H.; Roberts, C. A.; Wachs, I. E. Molecular Structural Determination of Molybdena in Different Environments: Aqueous Solutions, Bulk Mixed Oxides, and Supported MoO 3 Catalysts. *J. Phys. Chem. C* **2010**, *114* (33), 14110–14120. https://doi.org/10.1021/jp103269w.

(28) Thielemann, J. P.; Ressler, T.; Walter, A.; Tzolova-Müller, G.; Hess, C. Structure of Molybdenum Oxide Supported on Silica SBA-15 Studied by Raman, UV–Vis and X-Ray Absorption Spectroscopy. *Appl. Catal. A Gen.* **2011**, *399* (1–2), 28–34. https://doi.org/10.1016/j.apcata.2011.03.032.

(29) Thielemann, J. P.; Hess, C. Monitoring Silica Supported Molybdenum Oxide Catalysts at Work: A Raman Spectroscopic Study. *ChemPhysChem* **2013**, *14* (2), 441–447. https://doi.org/10.1002/cphc.201200648.

(30) Amakawa, K.; Sun, L.; Guo, C.; Hävecker, M.; Kube, P.; Wachs, I. E.; Lwin, S.; Frenkel, A. I.; Patlolla, A.; Hermann, K.; Schlögl, R.; Trunschke, A. How Strain Affects the Reactivity of Surface Metal Oxide Catalysts. *Angew. Chemie Int. Ed.* **2013**, *52* (51), 13553–13557. https://doi.org/10.1002/anie.201306620.

(31) Galeener, F. L.; Mikkelsen, J. C. Vibrational Dynamics in O 18-Substituted Vitreous Si O 2. *Phys. Rev. B* **1981**, *23* (10), 5527–5530. https://doi.org/10.1103/PhysRevB.23.5527.

(32) Vannice, M. A.; Joyce, W. H. *Kinetics of Catalytic Reactions*; Springer US: Boston, MA, 2005. https://doi.org/10.1007/b136380.

(33) Dumesic, J. A.; Rudd, D. F.; Aparicio, L. M.; Rekoske, J. E.; Trevino, A. A. *The Microkinetics of Heterogeneous Catalysis*, 1st Editio.; American Chemical Society, 1993.

(34) Gani, T. Z. H.; Berkson, Z. J.; Zhu, R.; Kang, J. H.; Di Iorio, J. R.; Chan, K. W.; Consoli, D. F.; Shaikh, S. K.; Copéret, C.; Román-Leshkov, Y. Promoting Active Site Renewal in Heterogeneous Olefin Metathesis Catalysts. *Nature* **2023**, *617* (7961), 524–528. https://doi.org/10.1038/s41586-023-05897-w.

(35) Chan, K. W.; Mance, D.; Safonova, O. V.; Copéret, C. Well-Defined Silica-Supported Tungsten(IV)–Oxo Complex: Olefin Metathesis Activity, Initiation, and Role of Brønsted Acid Sites. *J. Am. Chem. Soc.* **2019**, *141* (45), 18286–18292. https://doi.org/10.1021/jacs.9b09493.

(36) Freundlich, J. S.; Schrock, R. R.; Cummins, C. C.; Davis, W. M. Organometallic Complexes of Tantalum That Contain the Triamidoamine Ligand, [(Me3SiNCH2CH2)3N]3-, Including an Ethylidene Complex Formed via a Phosphine-Catalyzed Rearrangement of an Ethylene Complex. *J. Am. Chem. Soc.* **1994**, *116* (14), 6476–6477. https://doi.org/10.1021/ja00093a075.

(37) Hirsekorn, K. F.; Veige, A. S.; Marshak, M. P.; Koldobskaya, Y.; Wolczanski, P. T.; Cundari, T. R.; Lobkovsky, E. B. Thermodynamics, Kinetics, and Mechanism of (Silox) 3 M(Olefin) to (Silox) 3 M(Alkylidene) Rearrangements (Silox = t Bu 3 SiO; M = Nb, Ta). *J. Am. Chem. Soc.* **2005**, *127* (13), 4809–4830. https://doi.org/10.1021/ja046180k.

(38) Liu, S.; Boudjelel, M.; Schrock, R. R.; Conley, M. P.; Tsay, C. Interconversion of Molybdenum or Tungsten d 2 Styrene Complexes with d 0 1-Phenethylidene Analogues. *J. Am. Chem. Soc.* **2021**, *143* (41), 17209–17218. https://doi.org/10.1021/jacs.1c08086.
